# Interpreting comprehensive two-dimensional gas chromatography using peak topography maps with application to petroleum forensics

**DOI:** 10.1186/s13065-016-0211-y

**Published:** 2016-11-28

**Authors:** Hamidreza Ghasemi Damavandi, Ananya Sen Gupta, Robert K. Nelson, Christopher M. Reddy

**Affiliations:** 1 Department of Electrical Engineering, University of Iowa, 103 S Capitol Street, Iowa City, IA 52242 USA; 2 Department of Marine Chemistry and Geochemistry, Woods Hole Oceanographic Institution, 266 Woods Hole Road, Woods Hole, MA 02543 USA

**Keywords:** *GC* ×* GC*, Chromatography, Principal component analysis, Multivariate statistics, Quantitative interpretation, Oil-spill forensics

## Abstract

**Background:**

Comprehensive two-dimensional gas chromatography $(GC \times GC)$ provides high-resolution separations across hundreds of compounds in a complex mixture, thus unlocking unprecedented information for intricate quantitative interpretation. We exploit this compound diversity across the $(GC \times GC)$ topography to provide quantitative compound-cognizant interpretation beyond target compound analysis with petroleum forensics as a practical application. We focus on the $(GC \times GC)$ topography of biomarker hydrocarbons, hopanes and steranes, as they are generally recalcitrant to weathering. We introduce peak topography maps (PTM) and topography partitioning techniques that consider a notably broader and more diverse range of target and non-target biomarker compounds compared to traditional approaches that consider approximately 20 biomarker ratios. Specifically, we consider a range of 33–154 target and non-target biomarkers with highest-to-lowest peak ratio within an injection ranging from 4.86 to 19.6 (precise numbers depend on biomarker diversity of individual injections). We also provide a robust quantitative measure for directly determining “match” between samples, without necessitating training data sets.

**Results:**

We validate our methods across 34 $(GC \times GC)$ injections from a diverse portfolio of petroleum sources, and provide quantitative comparison of performance against established statistical methods such as principal components analysis (PCA). Our data set includes a wide range of samples collected following the 2010 *Deepwater* *Horizon* disaster that released approximately 160 million gallons of crude oil from the Macondo well (MW). Samples that were clearly collected following this disaster exhibit statistically significant match $(99.23 \pm 1.66 )\,\%$ using PTM-based interpretation against other closely related sources. PTM-based interpretation also provides higher differentiation between closely correlated but distinct sources than obtained using PCA-based statistical comparisons. In addition to results based on this experimental field data, we also provide extentive perturbation analysis of the PTM method over numerical simulations that introduce random variability of peak locations over the $(GC \times GC)$ biomarker ROI image of the MW pre-spill sample (sample $\#1$ in Additional file 4: Table S1). We compare the robustness of the cross-PTM score against peak location variability in both dimensions and compare the results against PCA analysis over the same set of simulated images. Detailed description of the simulation experiment and discussion of results are provided in Additional file 1: Section S8.

**Conclusions:**

We provide a peak-cognizant informational framework for quantitative interpretation of $(GC \times GC)$ topography. Proposed topographic analysis enables $(GC \times GC)$ forensic interpretation across target petroleum biomarkers, while including the nuances of lesser-known non-target biomarkers clustered around the target peaks. This allows potential discovery of hitherto unknown connections between target and non-target biomarkers.

**Electronic supplementary material:**

The online version of this article (doi:10.1186/s13065-016-0211-y) contains supplementary material, which is available to authorized users.

## Background

Comprehensive two-dimensional gas chromatography $(GC \times GC)$ provides high-resolution separation across hundreds, sometimes thousands, of crude oil hydrocarbons, thus unlocking unprecedented information for intricate quantitative interpretation. The broad objective of this work is to exploit this rich compound diversity and provide compound-cognizant quantitative interpretation of $(GC \times GC)$ peak topography that bridges the gap between target-driven analysis and statistical methods. We propose peak topography maps that extend individual $(GC \times GC)$ peak analysis beyond the well-known target peaks that dominate the $(GC \times GC)$ image, and present techniques for interpreting $(GC \times GC)$ topography that provide nuanced quantitative peak-based comparisons between $(GC \times GC)$ images. While we present our results in the context of petroleum forensics as a practical application of interest, the scope of our work applies generally to quantitative $(GC \times GC)$ interpretation and as such, goes beyond the stated application.

A key distinction of our technique against multi-variate statistical methods [1] is compound-cognizant interpretation that preserves the identity of individual target peaks while extending the scale of peak-level interpretation to all peaks, target and non-target, within the $(GC \times GC)$ topography. This allows nuanced $(GC \times GC)$ distinction between closely related yet different complex mixtures, e.g. crude oil from neighboring oil sources, which share the regional fingerprint, and therefore, difficult to differentiate robustly using purely statistical methods.

### Current state-of-the art in chromatographic interpretation: challenges and opportunities

Many separation technologies routinely filter out non-target analytes, thus eliminating possibility of understanding their connection to dominant target analytes in an environmental sample. More comprehensive data sets recording the joint contributions of target and non-target analytes may be enabled through comprehensive two-dimensional gas chromatography $(GC \times GC)$, liquid chromatography $(LC \times LC)$, mass spectrometry (MS) and combinations thereof. However, despite the informational richness of these comprehensive data sets, non-target analytes are traditionally ignored in sample analysis in preference to peak ratio comparisons between the target chemicals. Although non-target chemicals are empirically considered in the chemometric literature, their role is typically limited to the major statistical loadings in multi-variate distributions [2–4]. Thus, current state-of-the-art in environmental forensics and analytical chemistry are broadly divided into two complementary approaches:

- Target-based analysis [3–14]: Focuses on the target chemicals (well-known hopanes, steranes, diasteranes in petrochemicals) that dominate the analytical landscape as the major peaks in a chromatogram or a GC–MS image. This includes statistical methods employed towards target-based analysis [12, 15].
- Target-agnostic analysis [16–22]: Statistical pattern-recognition techniques that analyze comprehensive separation data sets using different forms of multi-variate analysis.

Additional file 2: Table S7 (in Section S7) provides a point-by-point comparison between the two approaches in the context of environmental forensics.

### Petroleum forensics using $GC\times GC$ separation of crude oil samples

**Fig. 1:**
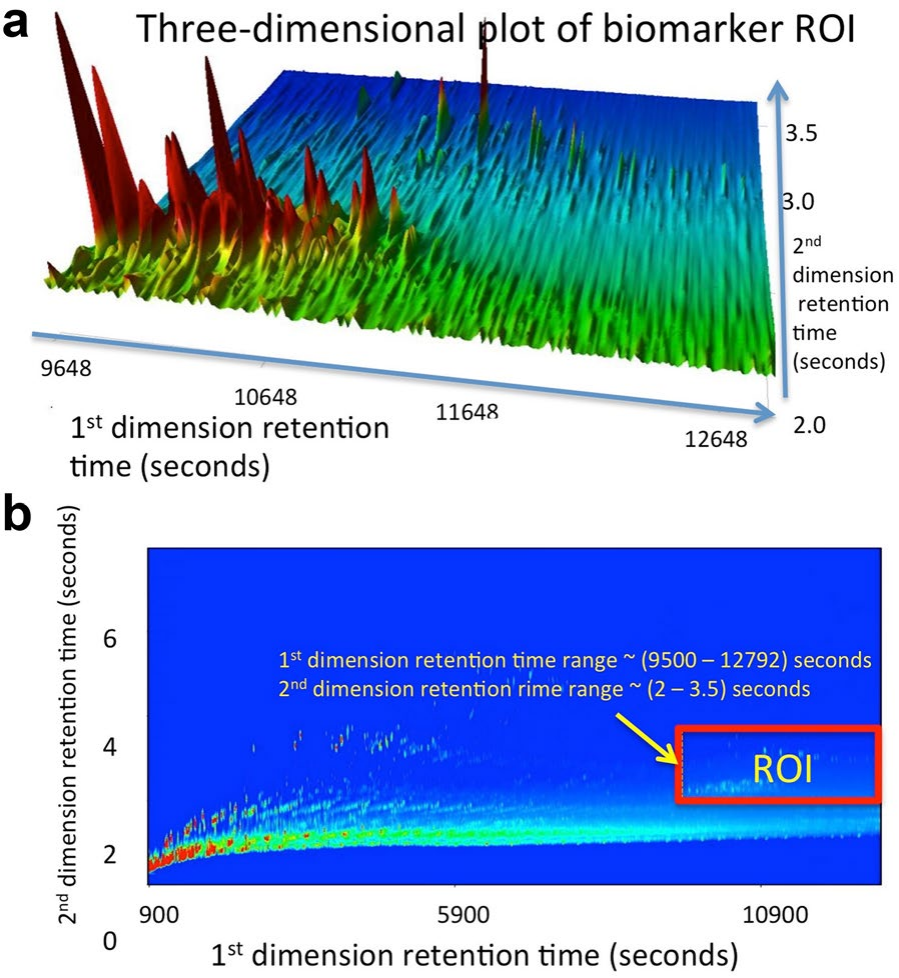
**a** The three-dimensional view of detailed topography of biomarker region (hopanes and steranes) within $GC\times GC$ image of crude oil pre-spill sample from MW, site of *Deepwater Horizon* spill disaster, Gulf of Mexico, 2010.** b** Biomarker region (hopanes and steranes) of (**a**) marked as the region of interest (ROI), shown as* red box* within full chromatogram.Target biomarkers within this ROI are labeled and itemized in Table S2. Total number of detected biomarker peaks (target and non-target) = 111, after removing peaks occupying lowest 5 % of the $GC\times GC$ peak magnitude profile as baseline noise. Range of considered peak summits (highest:lowest) = 14.53:1 (Aeppli et al. [25] Nelson et al. [36])

Reliable fingerprinting of petroleum and its weathered products has been an important field of study in the last four decades [2–10, 23–31]. Forensic analysis techniques fingerprinting crude oil samples in the ocean typically interpret the $GC\times GC$ peak profiles of biomarker hydrocarbons (hopanes and steranes), as they are generally recalcitrant against environmental weathering [4, 7, 11, 25–31]. Figure 1 shows the $GC\times GC$ hopane-sterane biomarker topography as the region of interest (ROI) within the full chromatogram of a pre-spill crude oil sample taken from the Macondo well (MW), source of the *Deepwater* *Horizon* disaster. The ROI biomarker region spans over a hundred compounds across a relative scale of 1−14.53 between the lowest and highest summits (peaks occupying lowest 5 % of the $GC\times GC$ peak magnitude profile were rejected as baseline noise). Traditional analysis employs approximately forty target biomarker compounds [refer to labeled compounds in Additional file 3: Table S2 (in Section S2)], which occur as major peaks dominating the $GC\times GC$ ROI biomarker topography, and about twenty well-known peak ratios [25] based on these target compounds.

### Background motivation: peak-cognizant interpretation beyond target biomarkers

Target biomarkers are generally abundant within a sample, robust to chromatographic variability, and therefore, provide a well-established basis to compare two oil samples [3, 4, 6, 7, 25]. However, the interpretation power of target analysis can be magnified significantly if we harness the full informational potential $GC\times GC$: combining the well-known characteristics of target biomarkers (major peaks) with the lesser known nuances of non-target biomarkers (minor peaks), which occupy the breadth of the intricate $GC \times GC$ topography. More recently, chemometric interpretations of $GC\times GC$ data sets have been proposed that adopt a multi-variate statistical approach to forensic interpretation [15–18, 32–34]. While these statistical approaches exploit the data variance of the $GC\times GC$ topography beyond the target peaks, they are typically agnostic of the target biomarkers and the dominant role they play in forensic interpretation [3, 4, 6–10, 25]. We harness the rich compound diversity across the $GC\times GC$ biomarker (hopanes and steranes) topography to provide potentially transformative compound-cognizant interpretation beyond target compound analysis.

Our objective is to extend the scope of target-centric standards [8–10] to include non-target biomarkers within a compound-cognizant framework, and thus bridge the gap between target-based forensics (e.g. [3, 4, 6, 7, 25] and references therein) and existing target-agnostic statistical approaches [15–17, 32–34]. We achieve source-specific and regional fingerprints by mapping connections between target and non-target biomarkers within the $GC\times GC$ topography. While the established target peaks dominate forensic interpretation, and can be individually identified in the topography map proposed, the unutilized contribution of the minor (non-target) peaks (e.g. the 73 unlabeled non-target peaks in Fig. 1) are also employed to distinguish closely related samples. Furthermore, we propose partitioning techniques that enable discovery of peak clusters connecting known targets to unknown non-target biomarkers, and thus derive common regional characteristics of petroleum-rich areas.

### Key innovation and contributions

Our motivation in this work is to achieve robust forensic distinction between closely related oil sources by utilizing rich peak information diversity in* GC* × *GC* chromatography. We validate our peak topographic methods across a set of 34 $GC\times GC$ injections from a diverse portfolio of petroleum sources, including a wide range of samples collected from the MW, the source of the *Deepwater Horizon* disaster in the Gulf of Mexico, April 2010. The MW samples exhibit statistically significant match $(99.23 \pm 1.66\%)$ against other closely related sources (Table 1). We introduce peak mapping and partitioning techniques that combine source-specific and regional characteristics manifested through the $GC\times GC$ topography of neighboring oil sources. We also provide a robust quantitative measure for directly determining “match” between samples, without necessitating training data sets. This is a key distinction against supervised learning techniques [19–22] that necessitate strong ground truths derived from large training databases that may be difficult to avail in the event of localizing a natural seep or surveying connectivity between newly discovered oil prospects. Our contribution is summarized in three novel concepts introduced in this work:

- Peak topography map (PTM), a feature representation that collectively captures $GC\times GC$ topography derived from the $GC\times GC$ chromatogram,
- Topography partitions, a threshold-based partitioning technique for discovering source-specific and regional characteristics, and
- Cross-PTM analysis, mathematical technique for directly determining “match” between two $GC\times GC$ separations without needing training data sets.

A natural outcome of PTM-based analysis is the discovery of topographic clusters (closely eluting groups of target and non-target biomarkers), which are key to understanding the regional and source-specific fingerprint.

**Table 1:** Percentage match (Mean ± standard deviation) between different Gulf of Mexico sources against MW injections for PTM with the optimal choice of peak ratio threshold $(\tau =1.65)$ and for PCA with two principal components

Method	MW (%) vs. MW (%)	Eugene Island (%) vs. MW (%)	Southern Louisiana Crude (SLC) (%) vs. (MW) (%)	Natural seep (%) vs. (MW) (%)
PTM	$99.23 \pm 1.66$	$85.83 \pm 3.70$	$59.28 \pm 13.34$	$52.55 \pm 1.94$
PCA	$99.76 \pm 0.26$	$91.98 \pm 0.14$	$91.71 \pm 0.24$	$98.01 \pm 0.51$

## Experimental

Additional file 4: Table S1 (in Section S1) lists the thirty-four injections along with the corresponding details on sample identity and geographic origin. The injections may be classified into three groups:

- Fourteen injections clearly originating from the MW, source of the *Deepwater Horizon* disaster;
- Three injections from non-Macondo well oil originating from three different sources in the Gulf of Mexico; and,
- Seventeen injections from diverse oil sources outside the Gulf of Mexico region.

In particular, injections 1 and 2 correspond to independent injections of a pre-spill sample taken directly from the MW during normal operations before the disaster; injection 3 corresponds to a surface slick sample from the MW collected after the spill; injection 4 is a post-spill sample collected directly from the broken riser pipe on June 21, 2010 [28, 35]; injections 5 through 14 correspond to ten separate oil samples that were obviously from the MW spill collected from grass blades along the Louisiana Gulf of Mexico coast; injections 15 and 16 are from two other crude oil sources from northern Gulf of Mexico and were collected before the *Deepwater Horizon* disaster, and injection 17 is collected from a natural oil seep in the Gulf of Mexico in 2006. The remaining injections correspond to distant sources unrelated to the Gulf of Mexico. For example, injections 18, 19 and 20 are independent consecutive injections of the National Institute of Standards and Technology (NIST) Standard Reference Material 1582 (its characteristics suggest it is derived from Monterey Shale and likely a California crude similar to injection 21).

### $GC\times GC$-flame ionization detector (FID) analysis

The samples were analyzed on a $GC\times GC$-FID system equipped with a Leco dual stage cryogenic modulator installed in an Agilent 7890A gas chromatograph configured with a 7683 series split/splitless auto-injector, two capillary columns, and a flame ionization detector. Samples were injected in splitless mode, and the split vent was opened at 1.0 minutes. The inlet temperature was 300 °C. The first-dimension column and the dual stage cryogenic modulator reside in the main oven of the Agilent 7890A gas chromatograph. The second-dimension column is housed in a separate oven installed within the main GC oven. With this configuration, the temperature profiles of the first-dimension column, dual stage thermal modulator, and the second-dimension column can be independently programmed. The first-dimension column was a Restek Rtx$^{-1}$, (30 m, 0.25 mm I.D., 0.25 $\upmu$m film thickness) that was programmed to remain isothermal at 45 °C for 10 min and then ramped from 45 to 315 °C at 1.2 °C min$^{-1}$. Compounds eluting from the first dimension column were cryogenically trapped, concentrated, and re-injected (modulated) onto the second dimension column. The modulator cold jet gas was dry nitrogen, chilled with liquid nitrogen. The thermal modulator hot jet air was heated to 45 °C above the temperature of the main GC oven (thermal modulator temperature offset = 45 °C). The hot jet was pulsed for 1.0 s every 12 s with a 5.0 s cooling period between stages. Second-dimension separations were performed on a SGE BPX50 (1 m, 0.10 mm I.D., 0.1 $\upmu$m film thickness) that remained at 75 °C for 10 min and then ramped from 75 to 345 °C at 1.2 °C min$^{-1}$. The carrier gas was hydrogen at a constant flow rate of 1.1 mL min$^{-1}$. The FID signal was sampled at 100 data points s$^{-1}$.

### Methods

We introduce the PTM representation of $GC\times GC$ data as an informational method that characterizes the peak information across the $GC\times GC$ biomarker topography as a connected graph. Wherever applicable in this work, peak refers to a single second-dimension peak, and $GC\times GC$ ROI refers to the biomarker sub-region (hopanes and steranes) of a two-dimensional gas chromatogram. Figure 1 illustrates this biomarker ROI within the full* GC* $\times$ *GC* chromatogram of the MW pre-spill sample listed as sample #1 in Table S1. We focus on the hopane-sterane biomarker topography as the region of interest (ROI) as these compounds are well-known for their recalcitrance to environmental degradation [25, 36].

#### Peak topography map (PTM) representation

PTM is a scalable node-based representation computed over a pre-selected $GC\times GC$ ROI representing the biomarker compounds. The PTM representation is scalable because: (i) PTM computation can be scoped to a smaller sub-region within the chosen $GC\times GC$ ROI, and (ii) PTMs computed across disjoint $GC\times GC$ ROIs can be combined to construct the PTM across the union of these regions, e.g. PTMs for the hopanes and steranes can be computed separated and then combined to give the PTM over both hopanes and steranes. Each PTM consists of a two-dimensional node structure that preserved peak characteristics, e.g. peak height, peak location and order of elution.

Mathematically, each peak collapses into a single PTM node that stores two attributes: (i) the magnitude at the peak summit, and (ii) peak location. We represent information at a PTM node (denoted as $\eta$) with the value assignment $\eta =\{p,m,n\}$, where *p* denotes the peak summit value, and *m* and *n* respectively denote the first and second dimension retention time indices for the particular peak in the $GC\times GC$ image.

The nodes are stored as an ordered two-dimensional matrix, with the first dimension coinciding with the first dimension retention time indices and the second dimension storing the PTM nodes in the consecutive order of elution of peaks along the second dimension. Thus the [*q*, *m*]-th element of the PTM matrix with node value $\eta =\{p,m,n\}$ stores the* q*th compound with peak height p, eluting along the second dimension with peak location [*n*, *m*] in the $GC\times GC$ image. The number of columns *N* of the PTM matrix represents the total number of first dimension modulations for the $GC\times GC$ ROI. The number of rows *Q* represents the maximum number of peaks eluting along the second dimension within the $GC\times GC$ ROI. The maximum number of peaks is computed across all second dimension indices within the $GC\times GC$ ROI. A PTM matrix column with fewer peaks than *Q* stores the PTM nodes in ascending order of peak locations, and populates the remaining entries with zeros to denote absence of a peak in those PTM nodes. We will henceforth refer to these entries in the PTM matrix that do not have a peak as “blank nodes”. To compute the PTM of a $GC\times GC$ ROI we normalize the PTM against the maximum value of the peaks. This normalization nullifies the effect of variable signal strengths between different injections by measuring all peak heights relative to the maximum signal strength within each $GC\times GC$ ROI. We locate all peaks within this ROI by employing a gradient-based maxima search (ref. Additional file 5: Section S4). Peaks that fall below $5\,\%$ of the maximum peak height within the $GC\times GC$ ROI are rejected as baseline noise. Mathematically, suppose the* n*th column of a $GC\times GC$ image has $\kappa _n$ number of peaks. The amplitudes and the locations of the peaks in this column can be stored in $Peak_n=\{p_{1,n},p_{2,n},\dots ,p_{\kappa _n,n}\}$ and $Loc_n=\{m_{1,n},m_{2,n},\dots ,m_{\kappa _n,n}\}$. We construct the $(l,n)$th element of its PTM representation matrix as:

1 $$\begin{aligned} PTM[l,n] = \left\{ \begin{array}{ll} p_{l,n}+j\times m_{l,n} &{} \text{ if } 1\le l \le \kappa _n \\ 0 &{} \text{ if } l > \kappa _n \end{array} \right. \end{aligned}$$

**Fig. 2:**
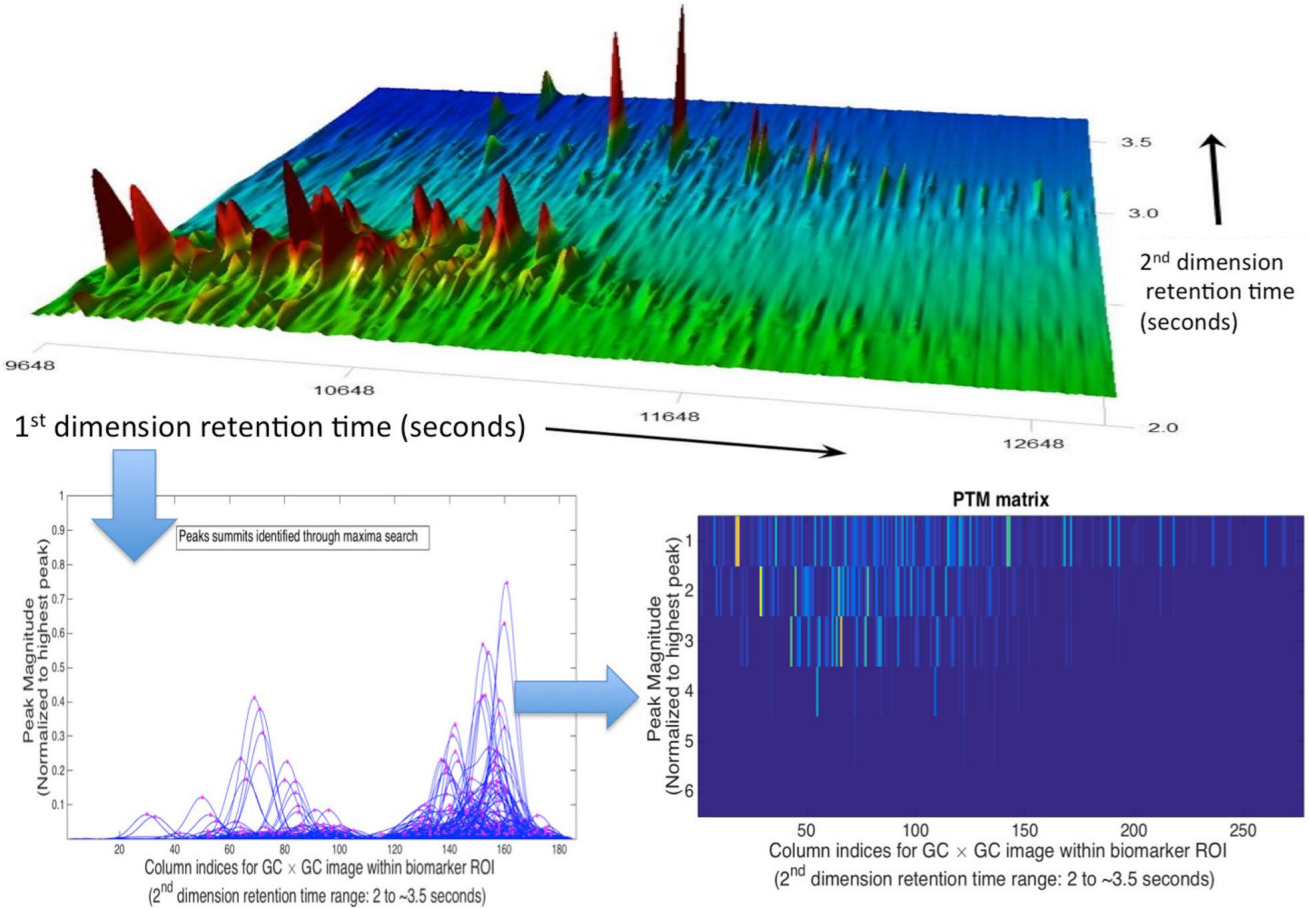
Step-by-step PTM construction Target biomarkers are labeled and itemized in Table S2. Total number of detected biomarker peaks (target and non-target) = 111, after removing peaks occupying lowest $5\,\%$ of the $GC\times GC$ peak magnitude profile as baseline noise. Range of considered peak summits (highest:lowest) = 14.53:1

**Fig. 3:**
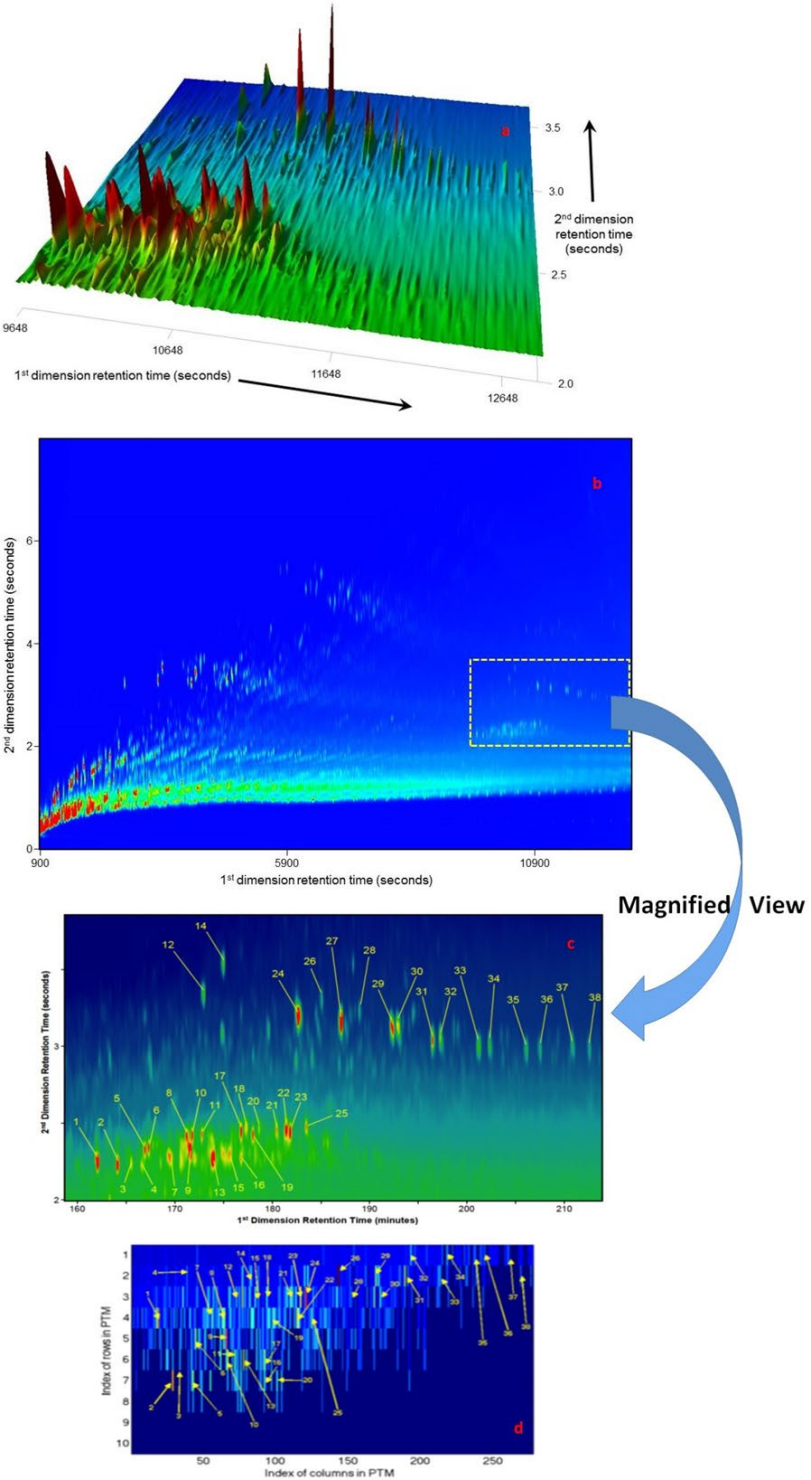
**a** The three-dimensional view of $GC\times GC$ image of crude oil sample from Eugene Island, Gulf of Mexico, about 50 miles southwest of MW, the oil source of the *Deepwater Horizon* disaster.** b** The two-dimensional view of the full chromatogram, with* yellow box* showing region of interest (hopanes and steranes) detailed in** a**.** c** Two-dimensional view of detailed topography of biomarker region (hopanes and steranes) marked as* yellow box* in** b**.Target biomarkers are labeled and itemized in Table S2.** d** PTM representation of ROI shown as* yellow box* in** b**. Thirty-eight target biomarkers are allocated to the numerically labeled PTM nodes. Each PTM node is uniquely assigned to each peak and therefore, each target peak is uniquely identifiable against the non-target peaks

In other words, if *l* corresponds to a peak location along the* n*th column of the $GC\times GC$ image, then the $(l,n)^{th}$ node of the PTM is a complex number with its real part as the amplitude of the peak and the imaginary part as its location. In case *l* does not correspond to a peak, (*l, n*)th node will be zero. Therefore, the problem of comparing two $GC\times GC$ image, like $I_{test}$ and $I_{ref}$ will turn into the problem of comparing the nodes at the same location in their PTM representation matrices. Figure 2 provides a visual representation of PTM computation for the crude oil MW pre-spill sample in Fig. 1 collected from the MW, Gulf of Mexico (injection 1 in Table S1). Figure 3 shows the full chromatogram, two and three-dimensional plots of the biomarker ROI and the PTM matrix corresponding to a sample from Eugene Island, another Gulf of Mexico source. The 38 target PTM nodes labeled for identification with the target compounds in the ROI biomarker region (detailed in Table S2) are highlighted in the constructed PTM matrix. *We note that the PTM matrices for the MW pre-spill sample (Fig.* 2) *and the Eugene Island sample (refer Fig.* 3) *are visually easier to distinguish than the original biomarker ROI image.*

Target compounds align according to their order of elution along the second dimension rather than absolute coordinates by design, thus rendering their location with respect to relative order of elution instead of specific retention times. Additional file 6: Algorithm 1 (in Section S3.1) and Additional file 7: Section S3.2 detail computational methods for ensuring PTM nodes compared across injections store the same compound within a pre-selected variability threshold. Local indexing of peak nodes with respect to relative order of elution instead of specific retention times makes the PTM interpretation robust to chromatographic variability within bounds $\{ \Theta _1, \Theta _2 \}$ (refer Additional file 6: Section S3, Algorithm 1) of expected variability selected by the user. Additional file 1: Section S8 and related discussion in the "Results" section also provides in-depth perturbation analysis of PTM interpretation against peak location variability. In summary, we observed that the PTM approach is relatively immune to variability even when introduced variability is greater than the bounds $\{ \Theta _1, \Theta _2 \}$ of expected variability selected by the user.

#### Topography partitioning: direct $GC\times GC$ comparisons based on aligned PTMs

We introduce topography partitioning as a visual quantitative informational method to facilitate direct comparison between two $GC\times GC$ ROIs. Topography partitions provide intricate cross-comparison between oil samples highlighting nuances of their biomarker topographies.

Topography partitions also form the basis for the cross-PTM score: a novel threshold-driven quantitative metric that provides a single numerical score for determining whether the two samples are a match. The key idea is to partition the $GC\times GC$ biomarker topography of a test sample based on which peaks, target and non-target, match against that of a reference sample using their respective PTM representations.

##### Mathematical computation of topography partitions

The peak-level match is determined using a peak ratio metric (ref. Equation S3.1 in Algorithm 1). This peak ratio metric is calculated at the granularity of individual PTM nodes and assessed against a pre-selected threshold to decide a match between the test and reference samples for a given compound. These individual match assessments are then conducted across peak profiles spanning the $GC\times GC$ ROI.

The topography is partitioned into “similar” and “dissimilar” peaks that meet or fall below the match threshold. The percentage of peaks in the “similar” topography generates the cross-PTM score. The two partitions are called similarity and dissimilarity partitions, where similarity indicates the partition of the test $GC\times GC$ ROI that matches that of the reference sample, and vice versa. Algorithm 1 provides a flowchart for determining the topography partitions of a test $GC\times GC$ ROI against a reference using PTM nodes.

In Additional file 6: Algorithm 1, Section S3.1, we have used a similarity criterion $\rho = max(a,a^{-1})$ where $\left(a = \frac{p_{ref}}{p_{test}}\right)$ is the peak ratio between two “equivalent” PTM nodes corresponding to the reference and test GC $\times$ GC ROIs. The notion of equivalence is determined by a user-constrained two-dimensional distance bound, denoted as $\{\Theta _1,\Theta _2\}$, between the two PTM node locations, as detailed in Step 1 of Additional file 6: Algorithm 1, Section S3.1. The function “$max(a,a^{-1})$” has a value greater than or equal to unity, with unity occurring when the peak heights $p_{ref}$ and $p_{test}$ exactly match. Generally due to baseline noise, column bleed and other chromatographic variability, the peak heights are not identical even if the GC $\times$ GC ROIs are created from the same oil source.

**Fig. 4:**
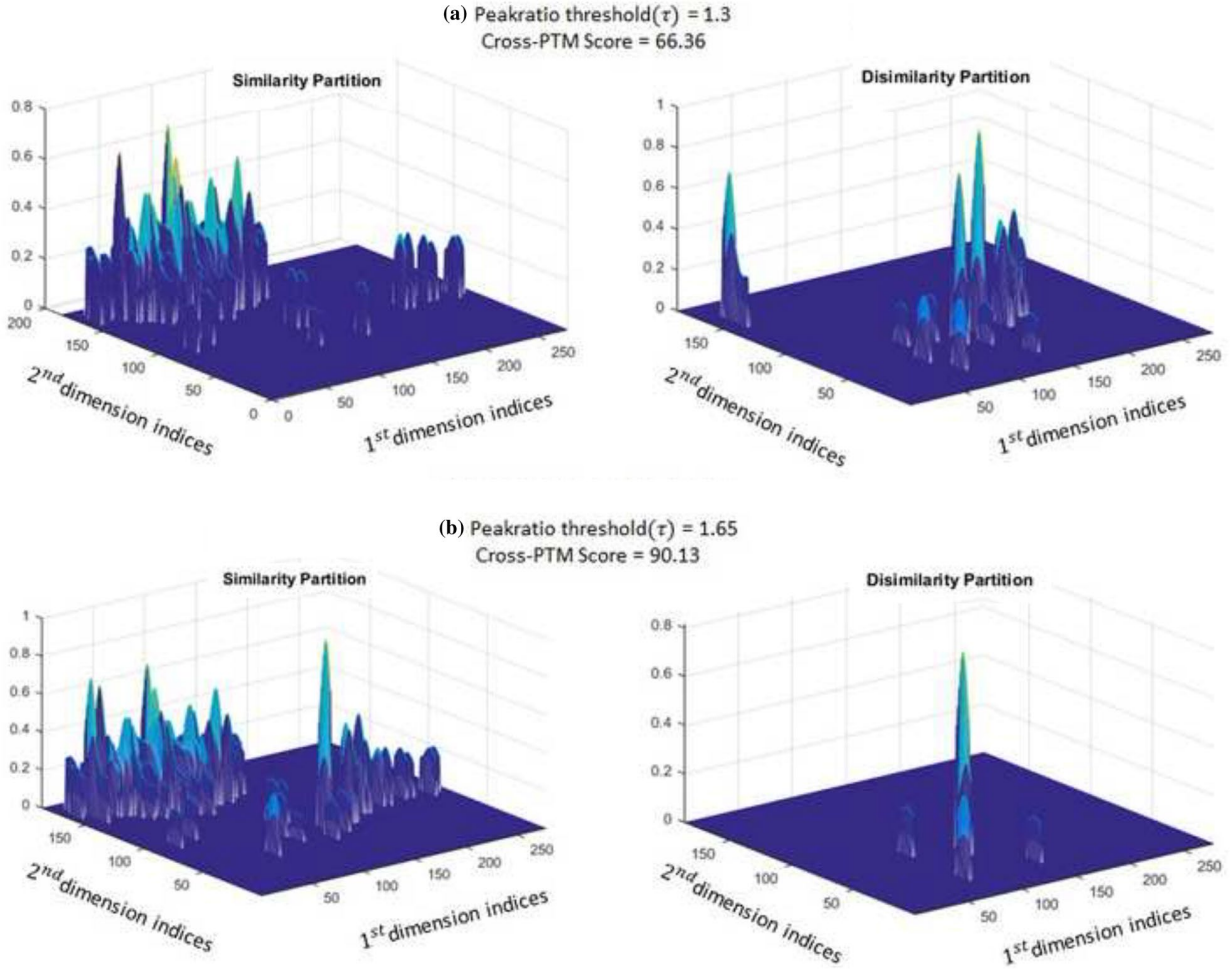
Topography partitioning of injection 15 (Eugene Island, Gulf of Mexico) with reference injection 4 (post-spill sample taken from the broken riser pipe of MW) for peak ratio threshold ** a** $\tau =1.3$ and** b** $\tau =1.65$

Therefore, we define a user-selected metric $\tau$ as a tolerance threshold and claim two peaks as “similar” if the function for those peaks is less than or equal to $\tau$ (e.g. in Table 1 the results are shown for $\tau = 1.65$). Figure 4 illustrates the topography partitions of two Gulf of Mexico injections, which originate in distinct sources, but share regional characteristics that are captured in the similarity partitions. Similarity partition represents the part of the GC $\times$ GC ROIs that exhibit “similar” peaks for a given $\tau$, and therefore, exhibit common characteristics between the $GC\times GC$ topography between the two injections. Alternatively, dissimilarity partition iterates the differences between the two $GC\times GC$ topographies. Therefore, topography partitions provide a threshold-dependent separation between the regional characteristics and source-specific features of a crude oil fingerprint. When the peak ratio threshold $\tau$ is increased, less peaks between the injections are classified as dissimilar, as evidenced in Fig. 4a and b. We now provide the mathematical representation for topographic partitions.

We denote the $GC\times GC$ ROI of the test and reference samples as $I_{ref}$ and $I_{test}$, the corresponding PTM matrices as $PTM_{test}$ and $PTM_{ref},$ and the PTM nodes as $\eta _{test}$ and $\eta _{ref}$ respectively. To compare the PTMs, we follow the algorithm detailed in Algorithm 1. We denote the modified $PTM_{test}$ after node insertions for alignment with $PTM_{ref}$ as $PTM_{test,aligned}(PTM_{ref}).$ The topography partitions are set up as a threshold classification of the test $GC\times GC$ ROI into two disjoint classes:

- Similarity partition: Portions of $I_{test}$ corresponding to test PTM nodes (originally present or inserted) that meet the peak ratio threshold $\tau$ (refer Step 3, Algorithm 1). We denote the similarity partition as $I_{test,similar}$.
- Dissimilarity partition: Portions of $I_{test}$ corresponding to test PTM nodes (originally present or inserted) that does not meet the peak ratio threshold $\tau$ (refer Step 3, Algorithm 1). We denote the dissimilarity partition as $I_{test,dissimilar}$.

We note that either partition not only includes the peak summits, but also the region under a peak. In the scenario where a node was inserted in the test PTM (refer Step 2b: Case 2, Algorithm 1) the $I_{test}$ partition will include the same peak sub-region corresponding to the equivalent peak region of $\eta _{ref}$, the reference PTM node.

##### Cross-PTM score calculation

The cross-PTM score, denoted as $S_\tau (I_{test}, I_{ref} )$, is a threshold-driven numerical comparison between the test and reference $GC\times GC$ ROIs that compares equivalent PTM nodes (refer Additional file 6: Section S3) for each ROI. Mathematically, it is derived as the weighted percentage of nodes in $PTM_{test, aligned}(PTM_{ref})$ that meet the threshold $\tau$ and therefore, belong in $I_{test, similar}$, i.e.,

2 $$\begin{aligned} S_\tau (I_{test}, I_{ref}) = \frac{|\eta _{test}\in PTM_{test,aligned}(PTM_{ref}):\rho (m,n) \ge \tau |_w}{|\eta _{test}\in PTM_{test, aligned}(PTM_{ref}) |} \end{aligned}$$

where $|\cdot |_w$ denotes the weighted sum taken across target and non-target peaks that meet the peak ratio threshold $\tau$ such that target (bigger) peaks are weighed higher than non-target (lower-valued) peaks. Additional file 8: Section S5 gives the detailed specification of weights as a function of peak heights used in this work. Figure 4 illustrates topography partitioning for injection 4 (MW post-spill sample) in Table S1 using injection 15 (from Eugene Island, Gulf of Mexico) as the reference for direct cross-PTM comparison for different thresholds. We note that the higher value of $\tau$ selects more of the topography into the similar partition, as is to be expected.

## Results and discussion

PTMs derived from $GC\times GC$ biomarker ROIs corresponding to 34 injections (refer Table S1 for details on origin) were compared pairwise against each other based on the threshold-based cross-PTM score. The 34 injections compared span across 31 distinct oil samples that originate from 19 distinct sources. Fourteen samples originate from the MW, source of the *Deepwater Horizon* disaster, including two pre-spill samples, and twelve post-spill samples collected at diverse locations after the *Deepwater Horizon* disaster, e.g. the plume at the base of the MW, grass blades on the Louisiana coastline, and oil slicks collected kilometers away from the disaster site (details provided in Table S1). These samples were collected in areas well documented [11, 25] to be heavily contaminated by the *Deepwater Horizon* disaster compared to the background.

We evaluate the cross-PTM score as a function of the peak ratio threshold across a diverse selection of injection pairs. We examine the robustness of intra-class match between injections of same origin against inter-class distinction between injection pairings from different origins. Specifically, we compare the fourteen MW injections (injections 1−14 in Table S1) against each other and against other sources within and outside the Gulf of Mexico region. We also compare the strength of MW vs. MW match against three other Gulf of Mexico injections (injections 15−17 in Table S1): (i) Eugene Island, (ii) Southern Louisiana Crude (SLC) and (iii) a Gulf of Mexico natural seep. Three consecutive injections from a non-Gulf of Mexico NIST sample originating in the Monterey area are also analyzed as an ideal intra-class case study, independent of any co-provenance bias with the Gulf of Mexico samples.

**Fig. 5:**
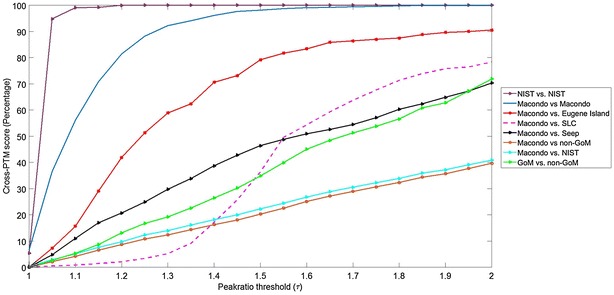
Mean cross-PTM scores plotted as a function of the peak ratio threshold $\tau$ for important intra-class (same source) and inter-class (distinct sources) comparisons. Each* plot* shows the average cross-PTM score taken over all possible pairings of injections for the corresponding comparison class (e.g. NIST vs. NIST plot shows the average cross-PTM score for three possible parings between the three NIST injections). Macondo refers to any crude oil sample originating from the MW, source of the *Deepwater Horizon* disaster

**Fig. 6:**
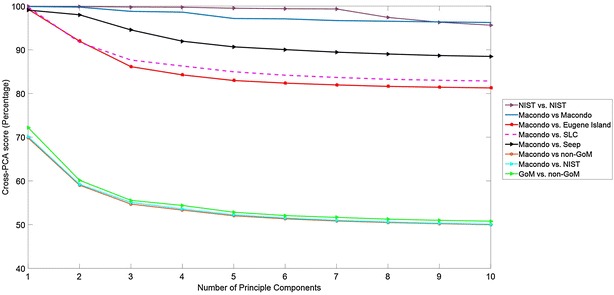
Mean cross-PCA scores plotted as a function of the peak ratio threshold $\tau$ for important intra-class (same source) and inter-class (distinct sources) comparisons. Each* plot* shows the average cross-PCA score taken over all possible pairings of injections for the corresponding comparison class (e.g. NIST vs. NIST plot shows the average cross-PCA score for three possible parings between the three NIST injections). Macondo refers to any crude oil sample originating from the MW, source of the *Deepwater Horizon* disaster

Figure 5 plots the average cross-PTM score as a function of peak ratio threshold across important comparison classes. Additional file 9: Figure S6.1 in Section S6 provides the statistical performance of the cross-PTM score for matching Gulf of Mexico injection pairs, with emphasis on distinguishing the 14 MW injections against non-Macondo Gulf of Mexico injections. We note that consistently the intra-class match between MW injections is statistically higher than the inter-class score between MW and other Gulf of Mexico injections. In Fig. 6, the cross-PCA score as a function of the number of principal components have been plotted. The statistical performance of the cross-PCA score for matching Gulf of Mexico injection pairs has been shown in Additional file 9: Figure S6.2 in Section S6.

### Best-case scenario for same-source match: NIST vs. NIST

To provide a neutral baseline for best-case performance, we compare three NIST injections (injections 19–21 in Table S1), all of which were taken from the same sample of non-Gulf of Mexico origin. The NIST injections were run consecutively under practically identical experimental conditions. We observe in Fig. 5 that the NIST vs. NIST cross-PTM score rapidly reaches $100\,\%$ match with increasing peak ratio threshold. This is to be expected as the $GC\times GC$ biomarker topographies of injections run consecutively from the same sample are expected to be very similar, if not identical. In reality, cross-comparisons for source determination are made between injections from different samples that may have same origin but are not consecutive runs from the same physical sample. $GC\times GC$ topographies for same-source injections from different samples are therefore, bound to exhibit more variation due to shifting of minor peaks, co-elution of different biomarkers, as well as baseline variability. Thus we expect the NIST vs. NIST cross-PTM performance to provide an idealized upper bound to measure cross-PTM score performance.

### Comparison between MW injections from fourteen distinct samples

The 14 MW injections exhibit a range of 105–131 detected peaks spanning target and non-target $GC\times GC$ biomarkers with highest-to-lowest peak ratio within an injection ranging from 14.27 to 16.22. Majority of the peaks considered are non-target biomarkers (only 38 target biomarkers present among over 100 biomarkers considered) and thus offer a nuanced cross-PTM interpretation that accounts for both target and non-target contributions to an oil fingerprint. From Table 1 we observe that the inter-class match between MW injection pairings is well within statistical range, i.e., within one standard deviation $(\sigma )$ of the statistical mean $(\mu )$, for robust $(\mu \pm \sigma )$ differentiation against other Gulf of Mexico injections.

Specifically, at the choice of $\tau =1.65$ the MW injections exhibit $(99.23 \pm 1.66\%$, Median:100 %) intra-class match, which is sufficient to distinguish against inter-class cross-PTM score with other Gulf of Mexico injections.

This choice of peak ratio, $\tau$, was empirically selected at $\tau = 1.65$ which was observed to give the best distinguishment between the MW and other Gulf of Mexico sources.

### Comparison between Gulf of Mexico injections and injections outside the region

We observe from Table 1 and Fig. 5 that using $(\mu \pm \sigma )$ differentiation the Gulf of Mexico injections are robustly differentiated against each other and also exhibit considerable distinction against sources outside the Gulf of Mexico region. In conclusion, we observe that the mean and median performance of the cross-PTM score is highly robust in source distinction and worst-case performance is sensitive to choice of peak ratio $\tau$ and number of detected peaks. Thus, the PTM approach combines target and non-target analysis to address multi-layered forensic questions regarding whether the injections are from the same sample, from different samples of same origin, from samples of different origin but similar locale, and so on as demonstrated above in our analysis based on a unique and diverse set of oil samples.

### Differentiation between PTM and PCA in scope and performance

As indicated earlier the proposed methods in chemometrics such as PCA can be applied towards quantitative $GC\times GC$ interpretation. However, purely statistical methods limit interpretation to peak aggregates, and as such, cannot provide peak-level interpretation. Therefore, by design PCA and similar multivariate statistical methods are compound-agnostic and cannot provide quantitative comparison based on relative compound concentrations in two complex mixtures. In particular, PCA analysis projects the $GC\times GC$ image along the main directions of data variance and therefore, is well-suited to application scenarios where the incentive is dimensionality reduction and compound-agnostic comparison between weakly correlated sources.

The primary aim of this work is to provide quantitative peak-level interpretation beyond target biomarkers, with the end goal of robust differentiation between petroleum sources that share regional commonalities, and therefore, have highly correlated $GC\times GC$ fingerprints. So, even minor nuances between two sources can carry important information to help us separate them once they are extracted from two closely located regions.

This differentiation between the two interpretation methods can be easily seen in Table 1, where we compare the best performance for differentiating between GoM oil sources using PTM and PCA cross-comparison scores. The optimal parameter choice for each method is provided (number of components for PCA and peak ratio threshold for PTM).

The intra-class match (MW vs. MW) is slightly higher using PCA than PTM but the inter-class differentiation (MW vs. other local sources) is significantly more robust using PTM over PCA. This is to be expected as PCA is biased towards the common regional fingerprint of the Gulf of Mexico locale, which constitutes the dominant component of data variance of $GC\times GC$ separations of crude oil collected in this region.

Mathematically, we can perform PCA cross-comparison between these correlated courses based on the non-dominant components, but these are typically vulnerable to baseline noise and other uncertainties, and as such, not reliable for robust source differentiation. This is evident in Fig. 6, where increasing the number of components increases gap between inter-class scores but also reduces the intra-class (MW vs. MW) match. On the other hand, cross-PTM match scores (Fig. 5) consistently provide high intra-class and considerably lower inter-class match scores over a wide range of the peak ratio threshold.

In summary, PCA enables statistical distinction between two $GC\times GC$ separations which have been extracted from geologically unrelated sources far apart from each other, but falls short of robust differentiation between strongly correlated sources located within the same region. PTM analysis provides peak-cognizant quantitative interpretation that can robustly differentiate between $GC\times GC$ separations between strongly correlated but distinct sources that share the regional fingerprint.

### Summary of Perturbation Analysis Based on Numerical Simulations

In addition to results based on this experimental field data, we also provide extensive perturbation analysis of the PTM method over numerical simulations that introduce random variability of peak locations over the GC$\times$GC biomarker ROI image of the MW pre-spill sample (sample $\#1$ in Table S1). We compare the robustness of the cross-PTM score against peak location variability in both dimensions and compare the results against PCA analysis over the same set of simulated images. Detailed description of the simulation experiment and discussion of results are provided in Additional file 1: Section S8. For the sake of completeness, we summarize below our main findings from the simulation experiment and reproduce some related discussion.

We observed that the PTM approach is relatively immune to variability even when introduced variability is greater than the bounds $\{ \Theta _1, \Theta _2 \}$ of expected variability selected by the user. Specifically, we observed that despite expected increase in intra-class (e.g. MW vs. MW) matching error as perturbation is increased, the inter-class match (e.g. Macondo vs. other Gulf of Mexico samples) scores nonetheless stays outside statistical bounds of an intra-class match. For example, increasing statistical perturbation of peak locations from five pixels to ten pixels in the second dimension and introducing perturbation by unit pixel in the first dimension reduces the inter-class (Macondo vs. Macondo) match between fifty simulated GC$\times$GC images against the template GC$\times$GC image (from pre-spill MW sample) from $100 \, \%$ (perturbation by only 5 pixels in second dimension) to $92\pm 5 \,\%$ match. However, the inter-class match scores (MW vs. other Gulf of Mexico samples from Eugene Island, Southern Louisiana and local natural seep) also change from $\{87.4\pm 1\,\%,47\pm 1\,\%,61.1\pm 1.8\,\%\}$ to $\{77.49\pm 4.4\,\%,39.8\pm 3.2\,\%,58.1\pm 1.47\,\%\}$. It is easy to see that despite the reduction in inter-class match due to increased perturbations, intra-class (MW vs. non-MW) match scores clearly fall outside the statistical $(\mu \pm \sigma )$ bounds of inter-class (MW vs. MW) match scores, where $\mu$ and $\sigma$ denote mean and standard deviation respectively.

In sharp contrast, the perturbation analysis of PCA scores over the same set of simulated images exhibit much higher “false alarm” match between classes, i.e., non-MW vs. MW comparisons. For example, the natural seep field sample was indistinguishable statistically from the Macondo class regardless of perturbation limits. PCA also exhibits much lower sensitivity to perturbations in the peak locations, which is to be expected, as it is a purely statistical compound-agnostic technique that does not consider one peak at a time. We note that the contrast between PCA and PTM observed over simulations is consistent with that observed over the field data.

## Ongoing research related to techniques proposed

The PTM method enables $GC\times GC$ forensic interpretation across well-known target biomarkers, while including the nuances of lesser-known non-target compounds clustered around the target peaks. This allows potential discovery of hitherto unknown connections between biomarkers that are related through topographic similarity between samples. The method proposed in this work is designed towards peak-to-peak comparisons, where each peak is distinctly formed and uniquely compared between samples (refer Additional file 6: Algorithm 1, Section S3.1). Therefore, the PTM method presented here is limited it its cluster-level interpretation, i.e., treating groups of compounds as one feature manifold. Moreover, significant co-elution of smaller non-target peaks can lead to imprecise identification of cluster content and intra-cluster distributions using the peak-based PTM technique proposed here. Nonetheless, there is potential to extend the idea of peak topography mapping towards clustered interpretation, combining similar peak groups as one feature. Some exploratory research with preliminary results regarding clustered interpretation and feature compression using peak pattern maps and manifold clusters is reported in [37–39]. It is out of scope for this work to examine compound clustering behavior and patterns derived thereof in detail, and deeper investigations are ongoing on whether the PTM method can be extended as a robust technique for knowledge discovery at the cluster level.

We also wish to iterate that the PTM method has been developed in this work with recalcitrant biomarkers in mind. It certainly has the potential to apply beyond the recalcitrant hopane-sterane biomarker region, for other crude oils and distillates, by measuring changes in peak heights of the same compounds across samples collected at different times and locations. Ideally, we believe we will be able to quantify weathering processes, subtract them from the signal, and continue to make highly quantitative comparisons. However, such investigations warrant their own detailed study and as such, are outside the scope of this paper.

## Conclusions

We introduce three novel concepts in this work: (i) PTM, a feature representation that collectively captures the $GC\times GC$ topography, (ii) PTM-based topography partitions, a threshold-based visualization technique for direct cross-sample comparisons, and (iii) cross-PTM analysis technique based on a quantitative score and topography partitions. Specifically, we address the broader question of what aspects of two oil samples are similar, and where do they differ, based on the molecular fossil (biomarker) topography of their $GC\times GC$ separations. Our methodology provides a mathematical framework for quantitative visualization of $GC\times GC$ at the granularity of individual peaks across target and non-target compounds as well as groups of peaks connected by topographic proximity. Such multi-scale interpretation is enabled by the combination of individual peak ratio evaluation between equivalent nodes, topography partitioning, and cross-PTM score spanning the collective topography of $GC\times GC$ ROI. We have validated our methods against experimental field data containing a diverse portfolio of oil samples across the world, with particular emphasis on the MW well, the source of *Deepwater Horizon* disaster, as well as over extensive perturbation analysis using numerical simulations (Additional files 10, 11).

## Additional files

**Additional file 1: Section S8.** Perturbation analysis based on numerical simulations.

**Additional file 2: Section S7.** Comparison between two broad analytic approaches to environmental forensics.

**Additional file 3: Section S2.** List of hydrocarbon biomarkers labeled as targets in the manuscript.

**Additional file 4: Section S1.** Tables of injections and target biomarkers.

**Additional file 5: Section S4.** Peak detection using maxima search.

**Additional file 6: Section S3.1.** Algorithm for cross-PTM comparison.

**Additional file 7: Section S3.2.** Node alignment example using Algorithm 1 in Section S3.1.

**Additional file 8: Section S5.** Weighted sum used for running Eq. 3 over data.

**Additional file 9: Section S6.** Statistical boundaries for cross-comparison scores for PTM and PCA.

**Additional file 10: Section S9.** Quantitative measurement of pixel variability within MW and NIST field data.

**Additional file 11: Section S10.** Coelution of peaks.

## Abbreviations

*GC* ×* GC*: two-dimensional gas chromatography
ROI: region of interest
PTM: peak topography map
