# Supplementary material for: Interpreting comprehensive two-dimensional gas chromatography using peak topography maps with application to petroleum forensics
Source: Chem Cent J. 2016 Nov 28;10:75. doi: 10.1186/s13065-016-0211-y (PMC5125045; doi:10.1186/s13065-016-0211-y)
Supplement: Supplementary file 1 — Additional file 1: Section S8. Perturbation analysis based on numerical simulations. [file 13065_2016_211_MOESM1_ESM.pdf]

Section S8: Perturbation Analysis Based on Numerical Simulations

1 Simulation experimental setup

In this supplementary document, we provide numerical evaluation of performance of our proposed method as well as PCA (principal component analysis) under perturbations of the peak location. Specifically, we simulate nine sets of GC×GC images, with fifty simulated images in each set created using perturbations from a reference image. In each simulated image, the peak locations are perturbed from their original position in a reference field sample. We chose the Macondo pre-spill (sample # 1 in Table S1) as our reference field sample, and denote the perturbations as η_1 and η_2 along the first and second dimensions respectively. Therefore, a peak location at (x_1, x_2) in the reference field sample is perturbed to a randomized value of $((x_1 + \eta_1), (x_2 + \eta_2))$ for each simulation, and each simulation set consists of 50 independent perturbed images. The perturbations (η_1, η_2) are randomly selected from uniform distributions $\eta_1 \sim \Delta(c_1, c_2)$ and $\eta_2 \sim \Delta(d_1, d_2)$, where (c_1, c_2) and (d_1, d_2) represent the upper and lower bounds of each distribution.

For example, $\eta_2 \sim \Delta(1, 5)$ represents randomized perturbations to peak locations along the second dimension, the random perturbation value picked from a uniform distribution between 1 and 5. Thus the simulated peak will lie with uniform probability within a perturbation range of 1 and 5 pixels in the second dimension from its original location in the reference field image.

We have chosen higher bounds of perturbations for the second dimension than the first dimension as variability is typically higher along the second dimension than the first.

1.1 Choice of variability thresholds

Local indexing of peak nodes with respect to relative order of eluton instead of specufic retention times makes the PTM interpretation robust to chromatographic variability within bounds $\{\Theta_1, \Theta_2\}$ (refer Section S3, Algorithm 1) of expected variability selected by the user. We chose $\Theta_1 = 1$ and $\Theta_2 = 10$ throughout this simulation experiment, which also coincides with the values chosen for the field data analysis.

1.2 Notational clarification

We have used the notation (x_1, x_2) to denote first-dimensional retention time x_1 and second-dimensional retention time x_2. This is different from matrix notation of a two-dimensional image $I(x, y)$ where x denotes row, which corresponds to second dimension for a GC×GC image and y denotes column, which corresponds to first dimension for a GC×GC image.

2 Simulation Results for Perturbation Analysis

Table S8 provides the details of these simulation experiments with results rounded upto the second decimal place.

Table S8.1: Cross-PTM perturbation analysis based on Macondo pre-spill reference sample

Perturbation	Cross-PTM match statistics between simulated GC×GC images and select field samples from different sources			
	Macondo	EI	SLC	Seep
$\eta_2 \sim \Delta(1,5),\ \eta_1 = 0$	100	87.43 ± 1.1	46.97± 1.17	61.11± 1.78
$\eta_2 \sim \Delta(1,10),\ \eta_1 = 0$	98.19±1.04	83.56± 1.19	45.64± 1.48	58.14± 1.36
$\eta_2 \sim \Delta(1,15),\ \eta_1 = 0$	97.85± 1.28	82.28± 1.84	42.32± 2.05	56.58± 1.9
$\eta_2 \sim \Delta(1,20),\ \eta_1 = 0$	93.69± 3.17	77.19± 3.6	38.04± 5.75	58.47± 1.4
$\eta_2 \sim \Delta(1,5),\ \eta_1 \sim \Delta(1,2)$	92.05± 4.9	68.21± 6.35	35.2319± 4.80	58.46± 1.49
$\eta_2 \sim \Delta(1,10),\ \eta_1 \sim \Delta(1,2)$	87.26± 2.19	63.16± 2.32	28.86± 3.69	50.75± 2.22
$\eta_2 \sim \Delta(1,15),\ \eta_1 \sim \Delta(1,2)$	85.02± 6.00	70.26± 6.16	34.48± 4.17	56.4± 1.96
$\eta_2 \sim \Delta(1,10),\ \eta_1 \sim \Delta(0,1)$	92.05± 4.9	77.49± 4.37	39.8±3.21	58.09± 1.47
$\eta_2 \sim \Delta(0,20),\ \eta_1 \sim \Delta(0,4)$	80.09±1.72	56.06± 5.90	24.70±3.48	50.33±4.47

Table S8.2: PCA cross-comparison perturbation analysis based on Macondo pre-spill reference sample

Perturbation	PCA match statistics between simulated GC×GC images and select field samples from different sources			
	Macondo	EI	SLC	Seep
$\eta_2 \sim \Delta(1,5),\ \eta_1 = 0$	99.96 ± 0.02	92.04± 0.02	91.48± 0.03	99.09± 0.04
$\eta_2 \sim \Delta(1,10),\ \eta_1 = 0$	99.62± 0.24	92.16± 0.10	91.61± 0.12	99.3± 0.11
$\eta_2 \sim \Delta(1,15),\ \eta_1 = 0$	99.45± 0.25	92.35± 0.17	91.80± 0.2	99.15± 0.15
$\eta_2 \sim \Delta(1,20),\ \eta_1 = 0$	99.04± 0.53	92.58± 0.22	92.05± 0.22	98.93± 0.19
$\eta_2 \sim \Delta(1,5),\ \eta_1 \sim \Delta(1,2)$	99.93± 0.03	92.36± 0.17	91.79± 0.18	99.02 ±0.06
$\eta_2 \sim \Delta(1,10),\ \eta_1 \sim \Delta(1,2)$	99.61± 0.24	92.61± 0.20	92.12± 0.21	99.17± 0.03
$\eta_2 \sim \Delta(1,15),\ \eta_1 \sim \Delta(1,2)$	99.8± 0.11	92.48± 0.21	91.90± 0.21	99.02± 0.05
$\eta_2 \sim \Delta(1,10),\ \eta_1 \sim \Delta(0,1)$	99.7± 0.18	92.38± 0.11	91.84± 0.13	99.31± 0.09
$\eta_2 \sim \Delta(0,20),\ \eta_1 \sim \Delta(0,4)$	99.48± 0.28	92.65± 0.19	92.12± 0.16	99.25± 0.08

We observe that the PTM approach is relatively immune to variability even when introduced variability is greater than the bounds $\{\Theta_1, \Theta_2\}$ of expected variability selected by the user. Specifically, we observe that despite expected increase in intra-class (e.g. Macondo vs. Macondo) matching error as perturbation is increased, the inter-class match (e.g. Macondo vs. other Gulf of Mexico samples) scores nonetheless stays outside statistical bounds of an intra-class match. For example, increasing statistical perturbation of peak locations from five pixels to ten pixels in the second dimension and introducing perturbation by unit pixel in the first dimension reduces the inter-class (Macondo vs. Macondo) match between fifty simulated GC×GC images against the template GC×GC image (from pre-spill Macondo sample) from 100% (perturbation by only 5 pixels in second dimension) to $92 \pm 5\%$ match. However, the inter-class match scores (Macondo vs. other Gulf of Mexico samples from Eugene Island, Southern Louisiana and local natural seep) also change from $\{87.4 \pm 1\%, 47 \pm 1\%, 61.1 \pm 1.8\%\}$ to $\{77.49 \pm 4.4\%, 39.8 \pm 3.2\%, 58.1 \pm 1.47\%\}$. It is easy to see that despite the reduction in inter-class match due to increased perturbations, intra-class (Macondo vs. non-Macondo) match scores clearly fall outside the statistical ($\mu\pm\ \sigma$) bounds of inter-class (Macondo vs. Macondo) match scores, where μ and σ denote mean and standard deviation respectively.

In sharp contrast, the perturbation analysis of PCA scores over the same set of simulated images exhibit much higher "false alarm" match between classes, i.e., non-Macondo vs. Macondo comparisons. For example, the natural seep field sample was indistinguishable statistically from the Macondo class regardless of perturbation limits. PCA also exhibits much lower sensitivity to perturbations in the peak locations, which is to be expected, as it is a purely statistical compound-agnostic technique that does not consider one peak at a time. We note that the contrast between PCA and PTM observed over simulations is

consistent with that observed over the field data.

3 Summary

We provide extensive statistical perturbation analysis through numerical simulations that statistically introduce higher variability of peak locations across both dimensions of the comprehensive two-dimensional gas chromatography (GC×GC) image. Specifically, we provide numerical evidence on that the source differentiation performance of our method is robust to perturbations in peak locations due to chromatographic variability, with more immunity towards second-dimensional variability than first-dimensional variability.
