# Supplementary material for: Interpreting comprehensive two-dimensional gas chromatography using peak topography maps with application to petroleum forensics
Source: Chem Cent J. 2016 Nov 28;10:75. doi: 10.1186/s13065-016-0211-y (PMC5125045; doi:10.1186/s13065-016-0211-y)
Supplement: Supplementary file 2 — Additional file 2: Section S7. Comparison between two broad analytic approaches to environmental forensics. [file 13065_2016_211_MOESM2_ESM.pdf]

Section S7: Comparison between two broad analytic approaches to environmental forensics.

Table S7: Comparison between two broad analytic approaches to environmental forensics.

**Comparison Metric**	**Target-based analysis (Peak ratio analysis between well-known analytes)**	**Target-agnostic analysis (Statistical pattern recognition)**
Focus of compound analysis	Focuses on individual nuances of well-known target compounds, which manifest as major peaks in chromatograms.	Focuses primarily on the statistical properties of the multi-variate chromatographic data.
Match criterion	Assign forensic interpretation based on the relative abundance of target biomarkers, typically using peak ratio measurements.	Forensic diagnosis based on large-scale empirical differentiations between the data distribution of specimens sampled from known sources.
Limitations of interpretation	Ignores the effect of (potentially hundreds of) non-target compounds, which occur in relatively minor proportions in the complex mixture	Does not distinguish between target (big peaks) and non-target compounds (minor peaks).
Immunity to retention time variability	Relatively immune to retention time variability of biomarker hydrocarbons, and robust across different crude oil specimens analyzed under diverse experimental conditions.	Vulnerable to retention time shifts which significantly shift the relative locations of minor peaks, and hence non-target compounds.
Contribution of individual compounds to source matching	Robust identification of target compounds that belong to source fingerprint.	Agnostic of forensic signatures of individual compounds.
Need for training data	Does not necessitate large-scale training data sets, few reliable source specimens may suffice.	Heavily dependent on training specimen libraries, reliably sampled from known source(s).
Reliability of source diagnosis	Provides reliable source diagnosis when the two sources exhibit distinct distributions across the major peaks.	Provides reliable source diagnosis when sufficient training samples are available to generate robust source ground truths.
Usage scenario	Best-suited for direct comparison between two or more specimens based on their target compound distribution.	Best suited for comparing samples with reliable ground truths (e.g. oil samples from industrial oil reservoirs, transformer storage sites vs. pigment manufacturing).

Academic Impact on oil-spill forensics	Essential for scientific understanding of well-known compounds in environmental forensics.	Essential for broad statistical distinction between well-known sources with reliable ground truths.
Practical impact on oil-spill forensics	Most chemists and EPA standards follow this approach due to higher scientific understanding and reliability of dominant compounds. Peak ratio analysis of target chemcials dominate forensic analysis of oil spills in environmental chemistry.	Most pattern-recognition techniques applied to oil-spill forensics fall in this domain, due to available pattern classification templates based on data statistics when reliable source ground truths are present.
