# Supplementary material for: Interpreting comprehensive two-dimensional gas chromatography using peak topography maps with application to petroleum forensics
Source: Chem Cent J. 2016 Nov 28;10:75. doi: 10.1186/s13065-016-0211-y (PMC5125045; doi:10.1186/s13065-016-0211-y)
Supplement: Supplementary file 3 — Additional file 3: Section S2. List of hydrocarbon biomarkers labeled as targets in the manuscript. [file 13065_2016_211_MOESM3_ESM.pdf]

Section S2: List of hydrocarbon biomarkers labeled as targets in the manuscript

Table S2: List of compounds labeled in Figures 1(c), 3(c) and 3(d)

Injection Number	Sample name	Sample description based on origin
1	DiaC27Ba-20S	13β(H),17α(H)-20S-diacholestane
2	DiaC27Ba-20R	13β(H),17α(H)-20R-diacholestane
3	DiaC27aB-20S	13α(H),17β(H)-20S-diacholestane
4	DiaC27aB-20R	13α(H),17β(H)-20R-diacholestane
5	DiaC28Ba-20S(24X)	24X-methyl-13β(H),17α(H)-20S-diacholestane
6	DiaC28Ba-20S(24Y)	24Y-methyl-13β(H),17α(H)-20S-diacholestane
7	DiaC28Ba-20R(24S&R)	24S&R-methyl-13β(H),17α(H)-20R-diacholestane
8	C27aBB-20R	5α(H),14β(H),17β(H)-20R-cholestane
9	DiaC29Ba-20S(24S&R)	24S&R-ethyl-13β(H),17α(H)-20S-diacholestane
10	C27aBB-20S	5α(H),14β(H),17β(H)-20S-cholestane
11	C27aaa-20R	5α(H),14α(H),17α(H)-20R-cholestane
12	Ts	18α(H)-22,29,30-trinorneohopane
13	DiaC29Ba-20R(24S&R)	24S&R-methyl-13α(H),17β(H)-20R-diacholestane
14	Tm	17α(H)-22,29,30-trinorhopane
15	DiaC29aB-20S(24S&R)	24S&R-ethyl-13α(H),17β(H)-20S-diacholestane
16	DiaC29aB-20R(24S&R)	24S&R-ethyl-13α(H),17β(H)-20R-diacholestane
17	C28aBB-20R	24-methyl-5α(H),14β(H),17β(H)-20R-cholestane
18	C28aBB-20S Unknown sterane mass 400	24-methyl-5α(H),14β(H),17β(H)-20S-cholestane
19	(C29)	Unknown sterane mass 400 (C29)
20	C28aaa-20R	24-methyl-5α(H),14α(H),17α(H)-20R-cholestane
21	C29aaa-20S	24-ethyl-5α(H),14α(H),17α(H)-20S-cholestane
22	C29aBB-20R	24-ethyl-5α(H),14β(H),17β(H)-20R-cholestane
23	C29aBB-20S	24-ethyl-5α(H),14β(H),17β(H)-20S-cholestane
24	NH	17α(H),21β(H)-30-norhopane

**Injection Number**	**Sample name**	**Sample description based on origin**
25	C29aaa-20R	24-ethyl-5α(H),14α(H),17α(H)-20R-cholestane
26	NM	17β(H),21α(H)-30-norhopane
27	H	17α(H),21β(H)-hopane
28	M	17β(H),21α(H)-hopane
29	HH(S)	17α(H),21β(H)-22S-homohopane
30	HH(R)	17α(H),21β(H)-22R-homohopane
31	2HH(S)	17α(H),21β(H)-22S-bishomohopane
32	2HH(R)	17α(H),21β(H)-22R-bishomohopane
33	3HH(S)	17α(H),21β(H)-22S-trishomohopane
34	3HH(R)	17α(H),21β(H)-22R-trishomohopane
35	4HH(S)	17α(H),21β(H)-22S-tetrakishomohopane
36	4HH(R)	17α(H),21β(H)-22R-tetrakishomohopane
37	5HH(S)	17α(H),21β(H)-22S-pentakishomohopane
38	5HH(R)	17α(H),21β(H)-22R-pentakishomohopane
