# Supplementary material for: Interpreting comprehensive two-dimensional gas chromatography using peak topography maps with application to petroleum forensics
Source: Chem Cent J. 2016 Nov 28;10:75. doi: 10.1186/s13065-016-0211-y (PMC5125045; doi:10.1186/s13065-016-0211-y)
Supplement: Supplementary file 4 — Additional file 4: Section S1. Tables of injections and target biomarkers. [file 13065_2016_211_MOESM4_ESM.pdf]

Section S1: Tables of injections and target biomarkers

All of the samples were collected without any necessary legal/operation permission or impacted endangered or protected species. However as part of the response to the Deepwater Horizon and a request from the official response, we collected the sample on June 21, 2010 at the Macondo well with assistance from the United States Coast Guard. This field sample is considered one of the most important from the Deepwater Horizon and eventually was involved in the Federal decision on the volume of oil released. Refer to [1, 2] for more information on its collection and usage in flow rate calculations. These samples were collected in areas well documented ([3, 4]) to be heavily contaminated by the Deepwater Horizon disaster compared to the background.

All of the other samples were collected by us out of scientific curiosity, obtained from colleagues, part of our in-house sample repository, or purchased.(references [1-4] below correspond to references 28, 35, 25 and 11 respectively, in the main manuscript.)

Table S1: List of Thirty-four injections across thirty-one samples from nineteen distinct sources

Injection Number	Sample name	Sample description based on origin
1,2	Macondo well oil	Sampled from the Macondo well before the Deepwater Horizon disaster, which occurred on April 20, 2010, as part of normal petroleum operations. It is often called the "pre-spill".
3	Surface sample	Oil droplet collected near the Deepwater Horizon blowout during the spill (June 2010)
4	Macondo well oil	Collected directly from the broken riser pipe at the Macondo well 6/21/2010. (Referred as MW-2 in Reddy et al 2011)
5	Grass blade-1	First distinct and separate sample scraped from one blade of marsh grass on May 30, 2010 about 200 km from the Deepwater Horizon blowout This sample and the following were clearly from the disaster based on tracking of surface slicks to this location.
6	Grass blade-2	Second distinct and separate sample scraped from one blade of marsh grass on May 30, 2010 about 200 km from the Deepwater Horizon blowout This sample and the following were clearly from the disaster based on tracking of surface slicks to this location.

7	Grass blade-3	Third distinct and separate sample scraped from one blade of marsh grass on May 30, 2010 about 200 km from the Deepwater Horizon blowout This sample and the following were clearly from the disaster based on tracking of surface slicks to this location.
8	Grass blade-4	Fourth distinct and separate sample scraped from one blade of marsh grass on May 30, 2010 about 200 km from the Deepwater Horizon blowout This sample and the following were clearly from the disaster based on tracking of surface slicks to this location.
9	Grass blade-5	Fifth distinct and separate sample scraped from one blade of marsh grass on May 30, 2010 about 200 km from the Deepwater Horizon blowout This sample and the following were clearly from the disaster based on tracking of surface slicks to this location.
10	Grass blade-6	Sixth distinct and separate sample scraped from one blade of marsh grass on May 30, 2010 about 200 km from the Deepwater Horizon blowout This sample and the following were clearly from the disaster based on tracking of surface slicks to this location.
11	Grass blade-7	Seventh distinct and separate sample scraped from one blade of marsh grass on May 30, 2010 about 200 km from the Deepwater Horizon blowout This sample and the following were clearly from the disaster based on tracking of surface slicks to this location.
12	Grass blade-8	Eighth distinct and separate sample scraped from one blade of marsh grass on May 30, 2010 about 200 km from the Deepwater Horizon blowout This sample and the following were clearly from the disaster based on tracking of surface slicks to this location.
13	Grass blade-9	Nineth distinct and separate sample scraped from one blade of marsh grass on May 30, 2010 about 200 km from the Deepwater Horizon blowout This sample and the following were clearly from the disaster based on tracking of surface slicks to this location.

14	Grass blade-10	Tenth distinct and separate sample scraped from one blade of marsh grass on May 30, 2010 about 200 km from the Deepwater Horizon blowout This sample and the following were clearly from the disaster based on tracking of surface slicks to this location.
15	Eugene Island crude	Collected from a drilling rig in the Eugene Island block 330, Gulf of Mexico.
16	Southern Louisiana Crude	SRM prepared by the US Environmental Protection agency (WP681). Collected in the 1970s from the Gulf of Mexico.
17	Gulf of Mexico seep	Natural oil seep (Collected in 2006 - 560 miles SW of the Deepwater Horizon disaster in the Gulf of Mexico).
18, 19, 20 (Injections from same sample analyzed consecutively)	NIST SRM-1582	Standard reference material (SRM) National Institute of Standards and Technology (NIST), likely from Monterey Shale.
21	Monterey crude	Crude oil collected off the coast of Santa Barbara, CA.
22	Kamchatka crude	Crude oil collected from Russia.
23	Ardjuna basin crude	Crude oil collected off the coast of Indonesia.
24	Exxon Valdez	Collected from the Exxon Valdez cargo after the March 1989 grounding.
25	Permean Basin	Crude oil collected in West Texas.
26	Arabian light crude	SRM prepared by the US Environmental Protection agency.
27	Kuwait export crude	First of three cargoes spilled from the MT Hebei Spirit (occurred 12/2007).
28	UAE - Upper Zakum crude	Second of three cargoes from the MT Hebei Spirit (occurred 12/2007).
29	Iranian heavy crude	Third of three cargoes from the MT Hebei Spirit (occurred 12/2007).
30	PetroEcuador crude	Crude oil collected off the coast of Ecuador.
31	Green River shale	Crude oil produced from the Green River Shale.
32	Texas crude	Collected from south central Texas.
33	Nigerian crude	Sample collected from Nigeria.
34	Angola Crude	Sample collected from Angola.

Author details

References

1. Reddy, C.M., Arey, J.S., Seewald, J.S., Sylva, S.P., Lemkau, K.L., Nelson, R.K., Carmichael, C.A., McIntyre, C.P., Fenwick, J., Ventura, G.T., *et al.*: Composition and fate of gas and oil released to the water column during the deepwater horizon oil spill. Proceedings of the National Academy of Sciences **109**(50), 20229–20234 (2012)
2. Camilli, R., Di Iorio, D., Bowen, A., Reddy, C.M., Techet, A.H., Yoerger, D.R., Whitcomb, L.L., Seewald, J.S., Sylva, S.P., Fenwick, J.: Acoustic measurement of the deepwater horizon macondo well flow rate. Proceedings of the National Academy of Sciences **109**(50), 20235–20239 (2012)
3. Aeppli, C., Nelson, R.K., Radovic, J.R., Carmichael, C.A., Valentine, D.L., Reddy, C.M.: Recalcitrance and degradation of petroleum biomarkers upon abiotic and biotic natural weathering of deepwater horizon oil. Environmental science & technology **48**(12), 6726–6734 (2014)
4. Aeppli, C., Carmichael, C.A., Nelson, R.K., Lemkau, K.L., Graham, W.M., Redmond, M.C., Valentine, D.L., Reddy, C.M.: Oil Weathering After the Deepwater Horizon Disaster Led to the Formation of Oxygenated Residues
