# Supplementary material for: Interpreting comprehensive two-dimensional gas chromatography using peak topography maps with application to petroleum forensics
Source: Chem Cent J. 2016 Nov 28;10:75. doi: 10.1186/s13065-016-0211-y (PMC5125045; doi:10.1186/s13065-016-0211-y)
Supplement: Supplementary file 5 — Additional file 5: Section S4. Peak detection using maxima search. [file 13065_2016_211_MOESM5_ESM.pdf]

Section S4: Peak Detection using Maxima search

Figure S4.1(a) is the three-dimensional plot of the GC×GC region of interest (ROI) representing the biomarker region within a larger GC×GC chromatogram of Macondo pre-spill sample #1 in Table S1. As stated in the manuscript, we employ a maxima finder using gradient computations to detect the peaks in a chromatogram. The key idea is to compute the gradient of the signal along the second dimension and locate the points where the gradient is zero with negative second derivative, indicating a maxima. Figure S4.1(b) demonstrates the results of the maxima finder over different peaks, major and minor, for the GC×GC ROI in Figure S4.1(a).

The peaks in Figure S4.1(b) have been plotted together as an overlaid collection. The overlaid visualization is intended to highlight the presence of hundreds of minor peaks besides the visible major peaks, and represents the same higher-dimensional information in the $GC \times GC$ image, and therefore, should not be treated as a collapsed GC plot. The range of second dimension retention times are given in the x-axis along with the pixel indices along each column of the GC×GC ROI image.

To provide resilience against noise, several measures may be taken to select a detected peak. We selected to keep the peaks detected by the maxima finder based on thresholding a ratio-driven measure. The metric chosen is sum of the absolute ratios of the slope to the peak width in the direction of rise and fall, as given in Equation S4. For the data analysis presented in Figure 3 in the manuscript, the peaks were thresholded to $\lambda \leq 0.01$. The variables used in Equation S4 below are defined in Figure S4.1(c).

$$\lambda = |\frac{s_1}{d_1}| + |\frac{s_2}{d_2}| \tag{1}$$

S4.1: Selection of the values of d1, d2 and the threshold for λ

Choice of d_1 and d_2 dictate the ratio $\lambda = |\frac{s_1}{d_1}| + |\frac{s_2}{d_2}|$, which jointly considers the four design parameters s_1, s_2, d_1 and d_2, where s_1 and s_2 are dependent on the choice of d_1 and d_2 and the peak maxima. Choosing too high a value for d_1 and d_2 can lead to erroneously counting several minor peaks as one major peak, and too low a value can lead to regarding noise bumps as peaks. Therefore, high values for d_1 and d_2 will also lower lambda and lead to higher vulnerability to noise if the lambda threshold is small, and the possibility of lumping several small peaks into one. Choosing too high a threshold for lambda leaves out many of the smaller minor compounds. To avoid these scenarios, we tested a range of values of d_1 and d_2 across a random sample of well-detected peaks across the chromatogram for several samples in our dataset. For simplicity, we chose $d_1 = d_2$ and $s_1 = s_2$. Based on our empirical observations, we chose the value to be $d_1 = d_2 = 5$, and lambda threshold to 0.001 in the cross-PTM analysis to best capture most of the topography without vulnerability to noise, and choose lower values of the parameters to highlight more minor peaks in Figure 1(c). For confirmation that the choice of the parameters captured most of the peaks in the dataset, we applied the peak detection algorithm to every chromatogram and generated Figure S4.1(b) for visual confirmation that all major peaks and considerable spread of minor peaks were detected.

S4.2: Baseline correction

The effect of column bleed, is compensated for using first-order interpolation between the "feet" of each peak. Figure S4.2 below shows the original and corrected baseline for one column within the $GC \times GC$ image.

Figure S4.1(a). Three-dimensional plot of $GC \times GC$ chromatogram region of interest (ROI) image of the sterane and hopane regions of a pre-spill crude oil sample from the Macondo well (sample 1 in Table S1).

Figure S4.1(b).Overlaid plot of peak shapes (in blue) and summit values (magenta stars on the peaks) detected along the 186 pixels along 2nd dimension for each of the 277 pixels in the 1st dimension of the $GC \times GC$ plot in Figure S4.1(a).

Figure S4.1(c). Peak parameters illustrated using a cosinusoidal peak.

Figure S4.2. Original and corrected baseline for one column within the GCxGC image. The baseline is corrected for column bleed by estimating the local column bleed using a simple linear estimator and subtracting its effect from the original curve. Visually speaking, this has the effect of calculating the local gradient between the "feet" (estimated using close-to-zero gradient search before and after the peak maxima) of the peak and then subtracting its effect from the original curve.

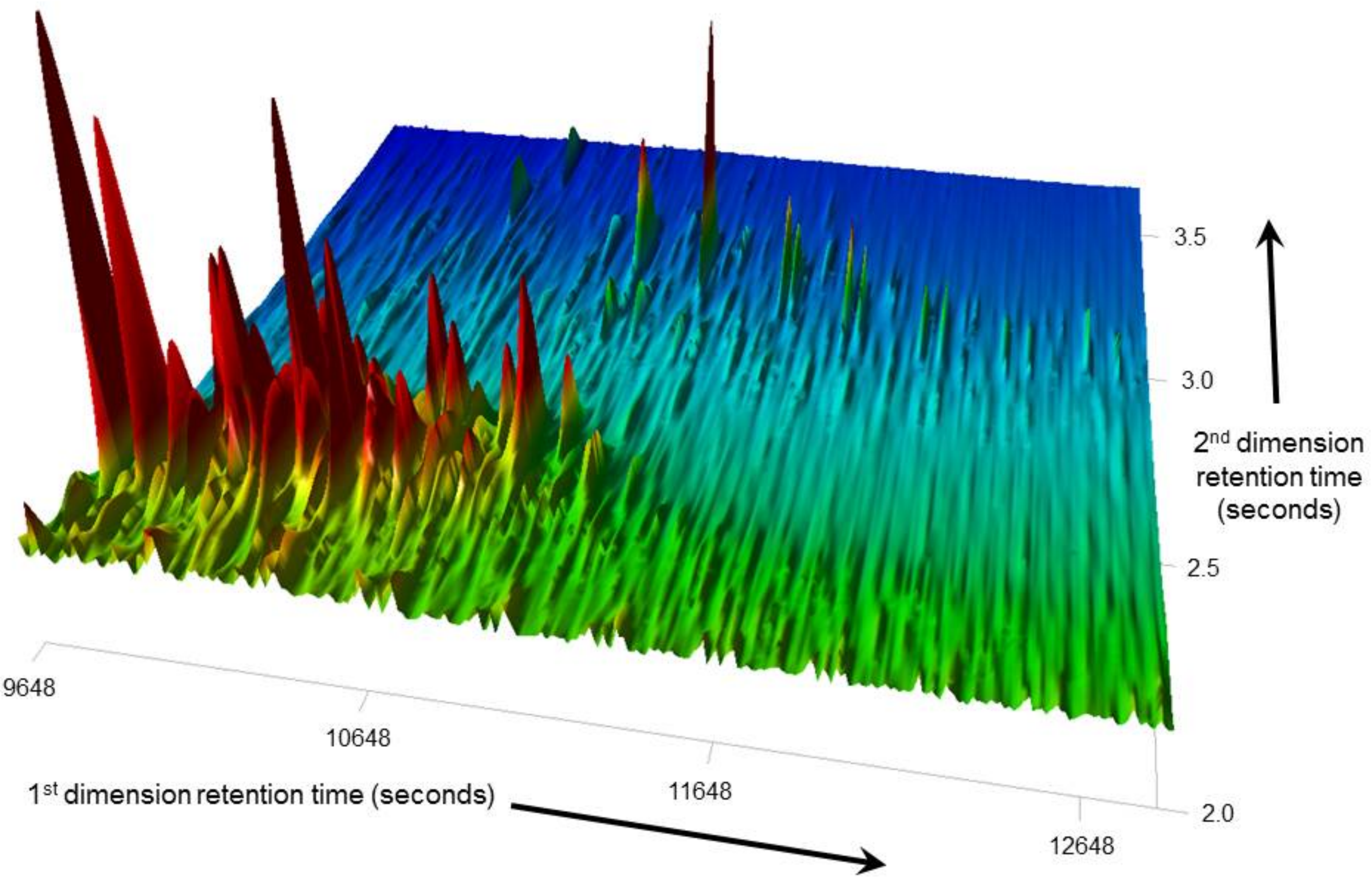
3.5
3.0
2.5
2.0
2nd dimension retention time (seconds)
9648
10648
11648
12648
1st dimension retention time (seconds)

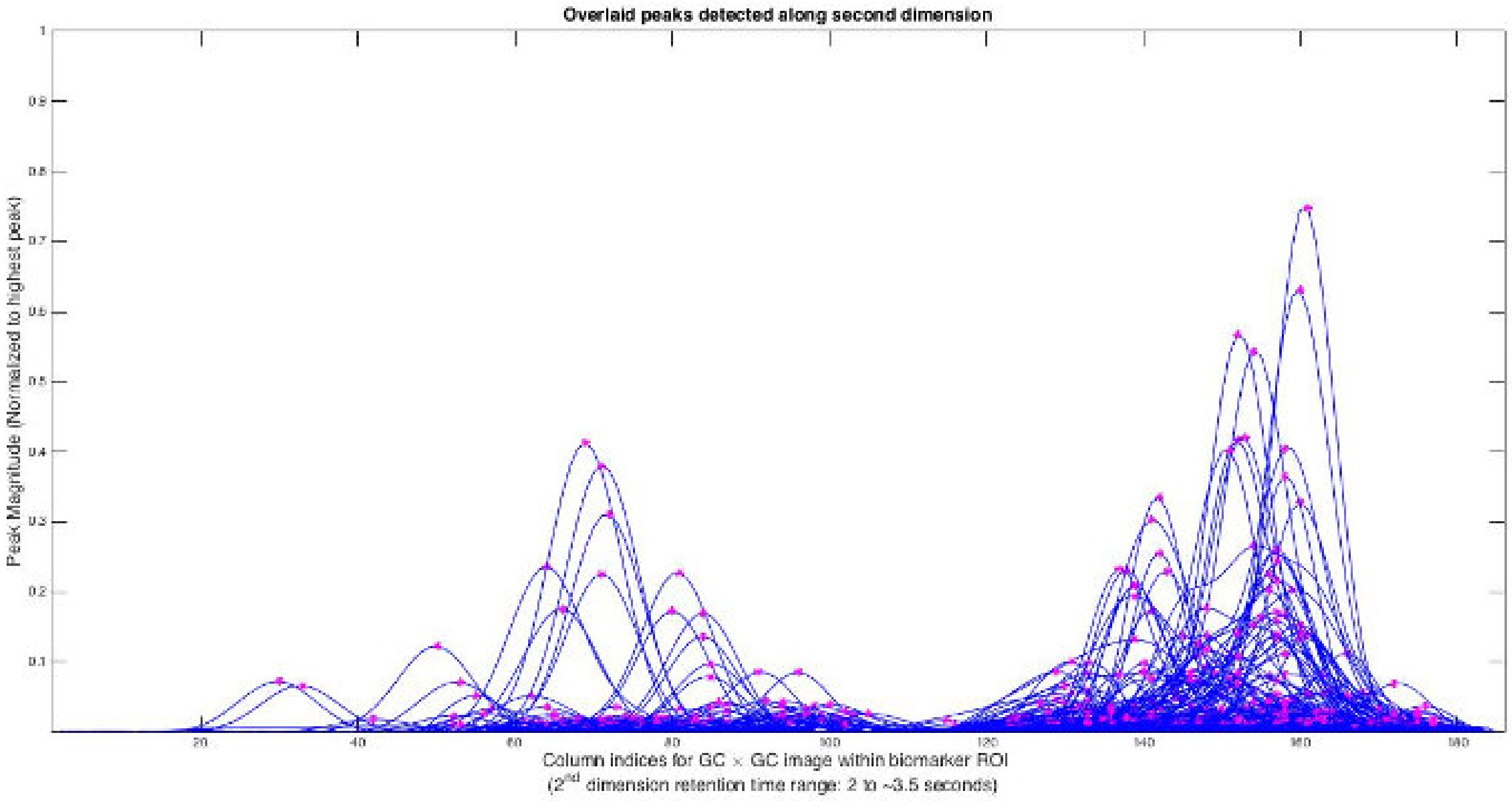
Overlaid peaks detected along second dimension
Peak Magnitude (Normalized to highest peak)
Column indices for GC × GC image within biomarker ROI
(2nd dimension retention time range: 2 to ~3.5 seconds)

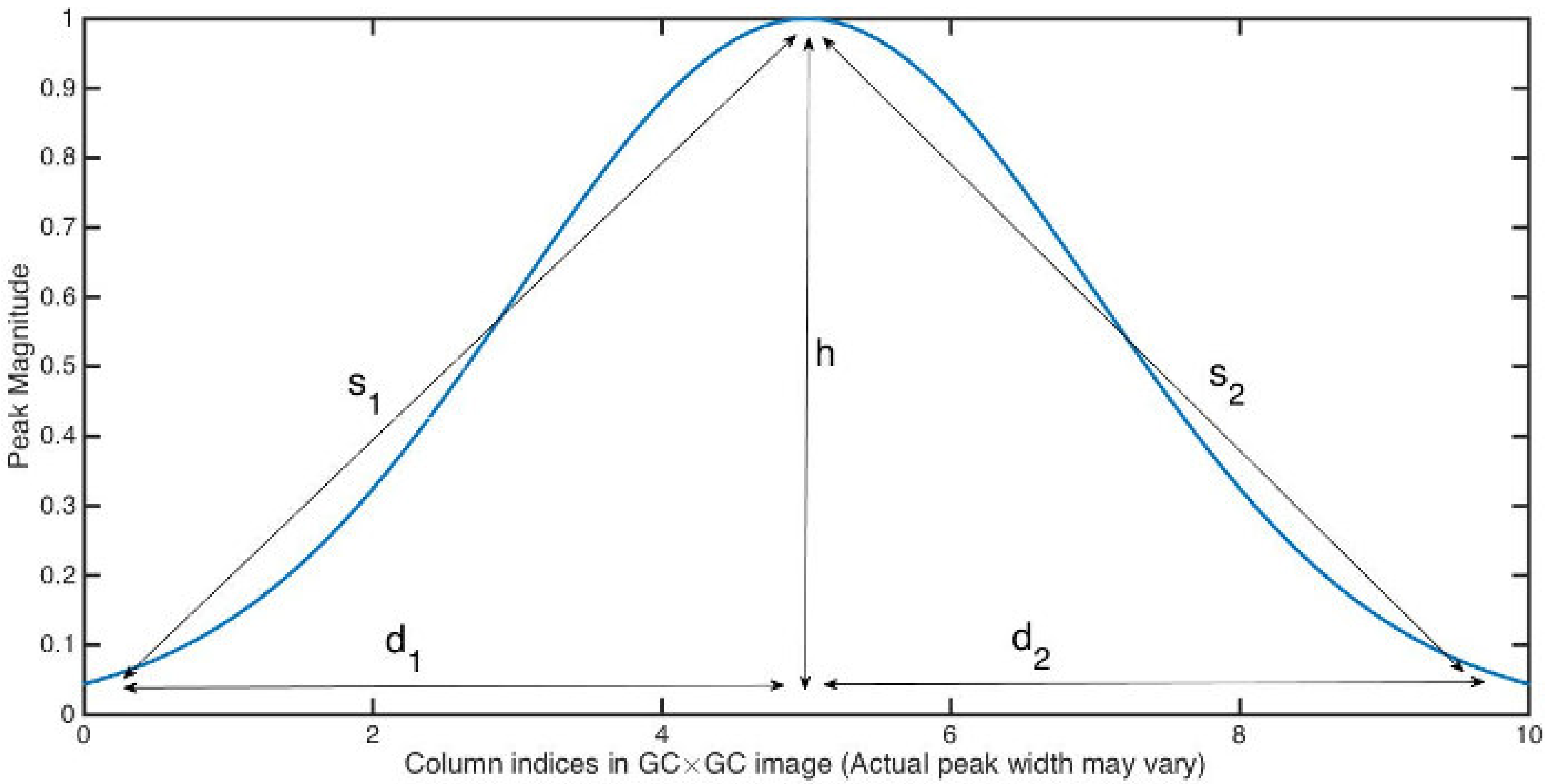

Peak Magnitude
Column indices in GC×GC image (Actual peak width may vary)
0
0.1
0.2
0.3
0.4
0.5
0.6
0.7
0.8
0.9
1
0
2
4
6
8
10
s1
s2
h
d1
d2

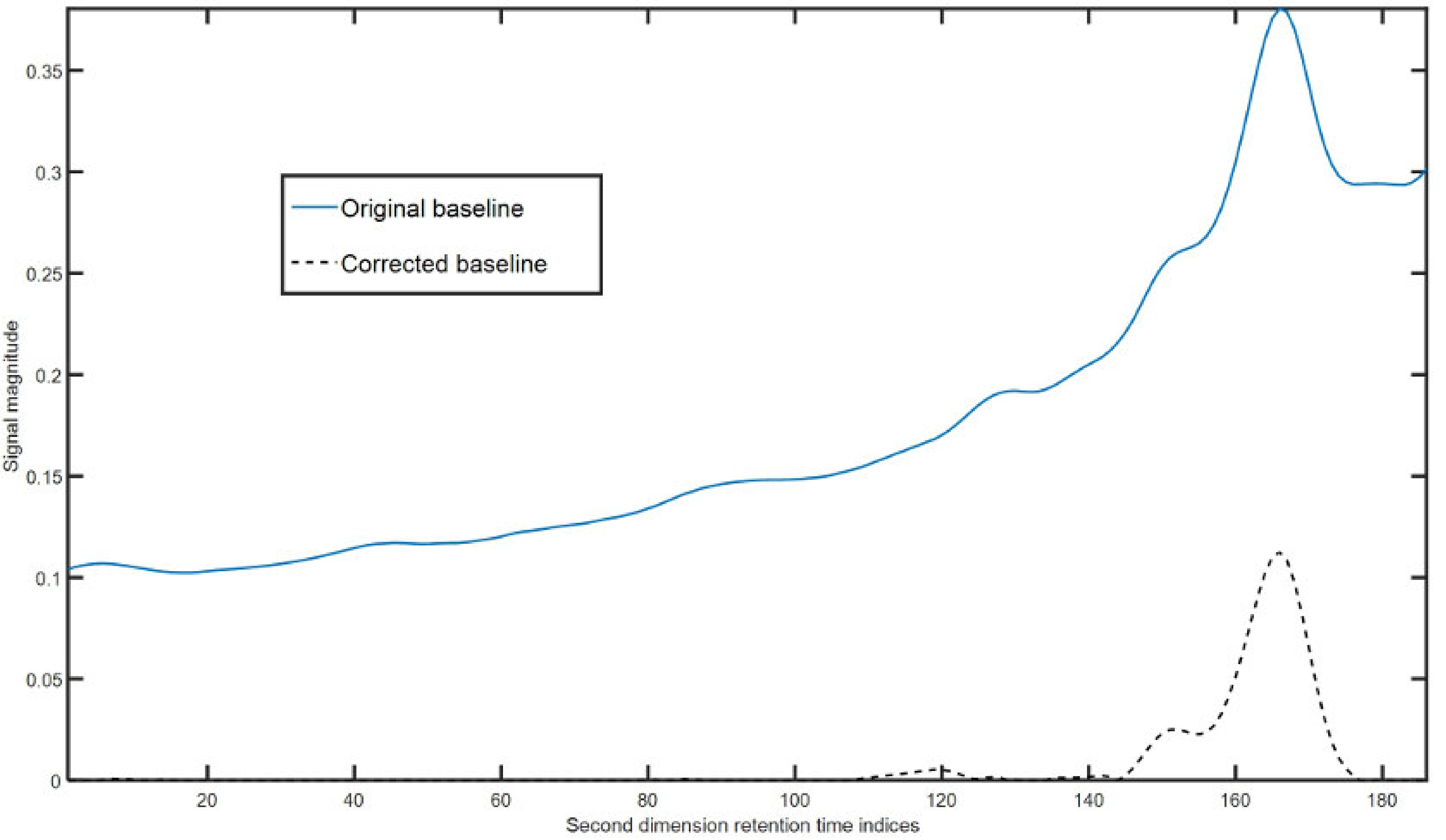

Original baseline
Corrected baseline
Signal magnitude
0
0.05
0.1
0.15
0.2
0.25
0.3
0.35
20
40
60
80
100
120
140
160
180
Second dimension retention time indices
