# Supplementary material for: Interpreting comprehensive two-dimensional gas chromatography using peak topography maps with application to petroleum forensics
Source: Chem Cent J. 2016 Nov 28;10:75. doi: 10.1186/s13065-016-0211-y (PMC5125045; doi:10.1186/s13065-016-0211-y)
Supplement: Supplementary file 6 — Additional file 6: Section S3.1. Algorithm for cross-PTM comparison. [file 13065_2016_211_MOESM6_ESM.pdf]

Section S3.1: Cross-PTM score computations

This section details the algorithm for computing the cross-PTM comparison score between two GC×GC images, represented by their PTM matrices. We note that the method does not put the peaks in the PTM matrix into bins, but rather, matches peak pairs between two samples locally within a region defined by the parameters $\{\Theta_1, \Theta_2\}$ around each peak. This renders the method robust against local shifts in peak locations due to chromatographic variability.

A step-by-step example of node alignment used in the cross-PTM algorithm is given in Section S3.2.

Notational clarification

We have used the notation (x_1, x_2) to denote first-dimensional retention time x_1 and second-dimensional retention time x_2. This is different from matrix notation of a two-dimensional image $I(x, y)$ where x denotes row, which corresponds to second dimension for a GC×GC image and y denotes column, which corresponds to first dimension for a GC×GC image.

Algorithm 1 Algorithm for cross-PTM comparison

Initialization:

Start at the top left node of each PTM matrix, i.e., choose $\eta_{test} = PTM_{test}[1, 1]$ and $\eta_{ref} = PTM_{ref}[1, 1]$.

Step 1:

Determine whether the PTM nodes to be compared are "equivalent", i.e., whether they contain the same compound, according to the following equivalence test: Let $\eta_{test} = \{m_1, n_1, p_{test}\}$ and $\eta_{ref} = \{m_2, n_2, p_{ref}\}$ denote the two PTM nodes being compared. We denote Θ_1 and Θ_2 as variability thresholds for the $GC \times GC$ ROI(region of interest refers to the biomarker sub-region (hopanes and steranes) of a two-dimensional gas chromatogram), which may be pre-selected locally for different groups of biomarkers. Consider the Euclidian metrics $\theta_1 = |m_1 - m_2|$, and $\theta_2 = |n_1 - n_2|$, which measure the distance between the PTM peaks along the first and second dimensions respectively. If $\theta_1 \leq \Theta_1$, and $\theta_2 \leq \Theta_2$, then the PTM nodes are considered equivalent.

If the nodes are equivalent, then proceed to Step 2a. If they are not equivalent, then proceed to Step 2b.

Step 2a:

Measure peakratio between equivalent nodes.

$$\rho(m_1, n_1) = \max\left(\frac{p_{ref}}{p_{test}}, \frac{p_{test}}{p_{ref}}\right) \quad (1)$$

where the peakratio is indexed by the location of the peak at the test PTM node.

Step 2b:

Determine the PTM node with the lower peak location in the second dimension, i.e., select η_{min}= $\arg_{\{n_1,n_2\}} min\{\eta_{test},\eta_{ref}\}$.

Step 2b - Case 1:($\eta_{min} = \eta_{test}$)

In this scenario, test $GC \times GC$ ROI has a peak at $[m_1, n_1]$ while the reference $GC \times GC$ has none within the (Θ_1, Θ_2)-neighborhood of $[m_1, n_1]$.
To compensate for the missing peak in the reference sample, we insert a new reference PTM node $\tilde{\eta}_{ref} = \{m_1, n_1, \tilde{p}_{ref}\}$ *preceding* the current reference node at η_{ref}=$\{m_2, n_2, p_{ref}\}$. We evaluate $\tilde{p}_{ref}$ as the maximum value within a (Θ_1, Θ_2)-vicinity of $[m_1, n_1]$ for the reference $GC \times GC$ ROI, i.e.,

$$\tilde{p}_{ref} = \arg\max I_{ref}\ (m_1 \pm \Theta_1, n_1 \pm \Theta_2) \quad (2)$$

The peakratio is evaluated as $\rho(m_1, n_1) = \max\left(\frac{\tilde{p}_{ref}}{p_{test}}, \frac{p_{test}}{\tilde{p}_{ref}}\right)$ between equivalent nodes η_{test}=$\{m_1, n_1, p_{test}\}$ and the inserted reference PTM node $\tilde{\eta}_{ref} = m_1, n_1, \tilde{p}_{ref}$. The peakratio is indexed by the location of the existing peak at the test node.

Step 2b - Case 2:($\eta_{min} = \eta_{ref}$)

In this other possible scenario, reference $GC \times GC$ ROI has a peak at $[m_2, n_2]$ while the test $GC \times GC$ ROI has none within the (Θ_1, Θ_2)-neighborhood of $[m_2, n_2]$. We insert a new test PTM node $\tilde{\eta}_{test} = \{m_1, n_1, \tilde{p}_{test}\}$ where $\tilde{p}_{test}$ denotes the maximum value within the (Θ_1, Θ_2)-neighborhood of the test $GC \times GC$ ROI, i.e.,

$$\tilde{p}_{test} = \arg\max I_{test}\ (m_2 \pm \Theta_1, n_2 \pm \Theta_2) \quad (3)$$

The peakratio is evaluated as $\rho(m_2, n_2)$= $\max\left(\frac{p_{ref}}{\tilde{p}_{test}}, \frac{\tilde{p}_{test}}{p_{ref}}\right)$ between the equivalent nodes η_{ref}=$\{m_2, n_2, p_{ref}\}$ and the inserted test PTM node $\tilde{\eta}_{test} = \{m_2, n_2, \tilde{p}_{test}\}$. In this case, the peakratio is indexed by the location of the existing peak at the reference PTM node.

Step 3:

We threshold the peakratio $\rho(m, n)$ indexed by the peak location at either or both PTM nodes by a pre-selected threshold τ. Each peak (in test sample, reference sample, or both) is classified as:

- "Similar" if $\rho(m, n) \leq \tau$, or
- "Dissimilar" if $\rho(m, n) \geq \tau$.

Step 4:

Increment the row index along the PTM matrix column (i.e., increment along the second $GC \times GC$ dimension) for the PTM node that did not have a node insertion. This reduces to three possibilities:

- Increment both PTM nodes for Step 2a,
- Increment test PTM node for Step 2b: Case 1, and
- Increment ref PTM node for Step 2b: Case 2.

Terminate and move to next PTM matrix column:

If both PTMs reach the last entry in the PTM matrix column, i.e, all remaining nodes in each PTM matrix column are blank nodes.
