# Supplementary material for: Interpreting comprehensive two-dimensional gas chromatography using peak topography maps with application to petroleum forensics
Source: Chem Cent J. 2016 Nov 28;10:75. doi: 10.1186/s13065-016-0211-y (PMC5125045; doi:10.1186/s13065-016-0211-y)
Supplement: Supplementary file 7 — Additional file 7: Section S3.2. Node alignment example using Algorithm 1 in Section S3.1. [file 13065_2016_211_MOESM7_ESM.pdf]

Section S3.2: Node alignment example using Algorithm 1 in Section S3.1

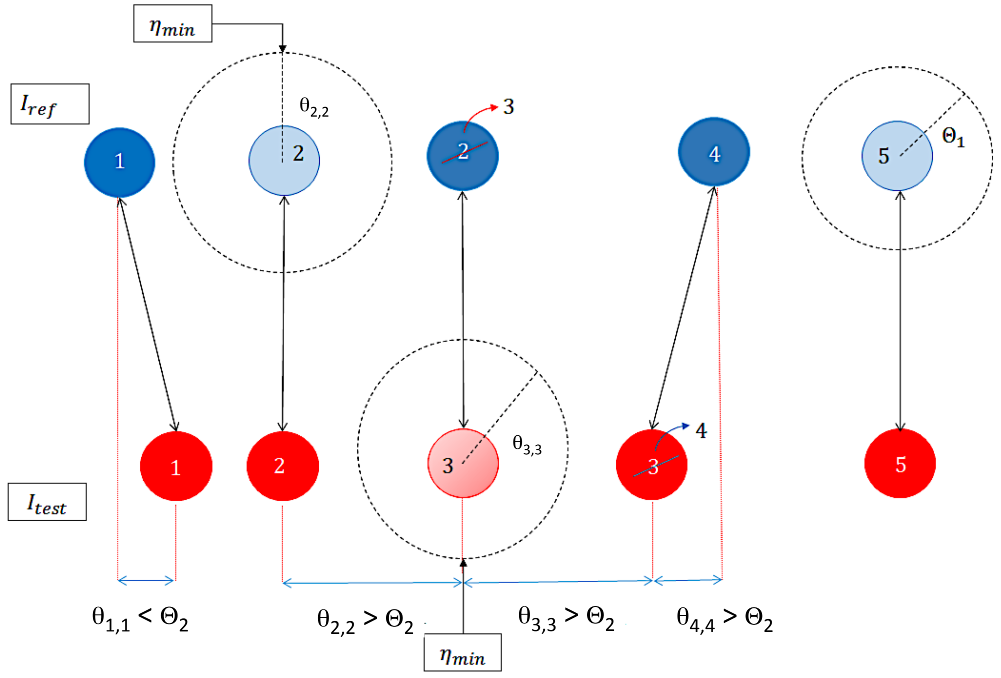

Figure S3.1. Visual step-by-step diagram of aligning PTM nodes between the PTM representation of two GC×GC ROIs from two samples.

Consider the PTM nodes for peaks along i^{th} column (i.e., second dimension), color-coded in blue for the reference image I_{ref}, and in red for the test (I_{test}) images. The reference image has four PTM nodes that correspond to peak summits identified through the gradient maxima search, and the test image has five nodes. We denote the distance along the second dimension between the mth PTM nodes, one from each image as $\theta_{m,m}$.

To align these PTM nodes so that they are considered as "equivalent" for Algorithm 1, i.e., are considered to represent the same compound, we proceed as follows. We also refer to Equations (2) and (3) from Algorithm 1, Section S3.1, as appropriate.

Step 1: We start with the first nodes of the both of the images, numbered 1 within the red and blue nodes. The distance between the locations of these two nodes are $\theta_{1,1}$ which is measured to be less than Θ_2 , Therefore, these two nodes are *already* aligned with each other and considered to be "equivalent" in Algorithm 1. We compare these two peaks to each other based on their peak ratio and proceed to the next nodes.

Step 2: We compute the distance between the second nodes of the images, numbered 2 with white color within the red and blue nodes.

The distance between them is $\theta_{2,2}$, which in this example is measured to be greater than Θ_1. Therefore, these two peaks are not equivalent and should not be mapped to each other. We

hold the peak with the lower location index, let's say η_{min} , and compensate for the missing peak by inserting a PTM node with the maximum amplitude around η_{min} with the radius of $\theta_{2,2}$ (refer Equation (2) in Algorithm 1). In Figure S3.1 above we compensate the missing peak node for the reference image at the location of η_{min} and insert the new PTM node as shown in light blue. The index of the compensated peak node in the reference image will be 2, and the index of the next node will change from 2 to 3 in the reference image, as shown above. The second nodes of the reference and test images are now aligned to each other, and we proceed to the next nodes.

Step 3: We compute the distance between the third nodes of the images, numbered 3 with white color within the red and PTM nodes.

The distance between them is $\theta_{3,3}$ which greater than Θ_2. These two peaks should not be mapped to each other. We hold the peak with the less location index, let's say η_{min} , and compensate the missing peak by inserting a peak node with the maximum amplitude around η_{min} with the radius of $\theta_{2,2}$ (refer Equation (3)). In Figure S3.1 we compensate the missing peak node for the test image at the location of η_{min}.

Now, the index of the compensated peak node in the test image will be 3, and the index of the next node will change from 3 to 4 in the test image, as shown above.

The third nodes of the reference and test images are mapped to each other now, we then proceed to the next nodes.

Step 4: The distance between the fourth locations of these two nodes are $\theta_{4,4}$ which is less than Θ_2. Therefore, these two nodes are mapped to each other. We map these two peaks to each other and proceed to the next nodes.

Step 5: The test image has the fifth peak node where this node does not exist in the reference image. We compensate the missing peak in the reference image by inserting a peak node with the maximum amplitude (Equation (2)) at the test peak node location with the radius of Θ_2. In the figure above we compensate the fifth missing peak node for the reference image.
