# Supplementary material for: Interpreting comprehensive two-dimensional gas chromatography using peak topography maps with application to petroleum forensics
Source: Chem Cent J. 2016 Nov 28;10:75. doi: 10.1186/s13065-016-0211-y (PMC5125045; doi:10.1186/s13065-016-0211-y)
Supplement: Supplementary file 8 — Additional file 8: Section S5. Weighted sum used for running Eq. 3 over data. [file 13065_2016_211_MOESM8_ESM.pdf]

Section S5: Weighted sum used for running Equation (3) over data

The following Matlab script is provided to document the weighted percentage we used for $| \cdot |_w$ in Equation (3) over our dataset to highlight difference in major peaks while accounting for minor peaks. The weights were determined empirically to provide greater weight to major peaks, which typically represent the target analytes, while considering the minor peaks, which typically represent non-target analytes. The minor peaks were grouped into different weight groups based on their relative heights. All peaks were normalized to the highest peak to mitigate the impact of variability between samples. We first determine the higher peak between the two peaks being compared, one from the test PTM node and the other from the reference PTM node, and then assign the weights based on the height of the higher peak.

Algorithm 1 function weighted$_{peaks} = weightedpeaks(peak1, peak2)$

$peak = max(peak1, peak2);$
if $(peak > 0.7)$ **then**
  $weighted_{peaks} = 10 * peak;$
end if
if $((peak > 0.5)\ \&\ (peak \leq 0.7))$ **then**
  $weighted_{peaks} = 10 * peak;$
end if
if $((peak > 0.3)\ \&\ (peak \leq 0.5))$ **then**
  $weighted_{peaks} = 3 * peak;$
end if
if $((peak > 0.2)\ \&\ (peak \leq 0.3))$ **then**
  $weighted_{peaks} = 0.5 * peak;$
end if
if $((peak > 0.1)\ \&\ (peak \leq 0.2))$ **then**
  $weighted_{peaks} = 0.001 * peak;$
end if
