# Supplementary material for: Interpreting comprehensive two-dimensional gas chromatography using peak topography maps with application to petroleum forensics
Source: Chem Cent J. 2016 Nov 28;10:75. doi: 10.1186/s13065-016-0211-y (PMC5125045; doi:10.1186/s13065-016-0211-y)
Supplement: Supplementary file 9 — Additional file 9: Section S6. Statistical boundaries for cross-comparison scores for PTM and PCA. [file 13065_2016_211_MOESM9_ESM.pdf]

Figure S6.1. Statistical comparison; $(\mu \pm \sigma)$, when μ denotes the means and σ denotes the standard deviation of cross-PTM match between Macondo and other Gulf of Mexico injections: Eugene Island, Southern Louisiana Crude (SLC) and Gulf of Mexico natural seep.

Figure S6.2. Statistical comparison; $(\mu \pm \sigma)$, when μ denotes the means and σ denotes the standard deviation of cross-PCA match between Macondo and other Gulf of Mexico injections: Eugene Island, Southern Louisiana Crude (SLC) and Gulf of Mexico natural seep.

Section S6: Statistical boundaries for Cross-comparison scores for PTM and PCA
