# Supplementary material for: Interpreting comprehensive two-dimensional gas chromatography using peak topography maps with application to petroleum forensics
Source: Chem Cent J. 2016 Nov 28;10:75. doi: 10.1186/s13065-016-0211-y (PMC5125045; doi:10.1186/s13065-016-0211-y)
Supplement: Supplementary file 10 — Additional file 10: Section S9. Quantitative measurement of pixel variability within MW and NIST field data. [file 13065_2016_211_MOESM10_ESM.pdf]

Section S9: Quantitative measurement of pixel variability

We have provided quantitative estimates of the degree of variability for the source of interest (i.e. Macondo) across the dataset in the revised manuscript and related supplemental documentation. This is summarized in Figure S9, which is reproduced here for convenience.

Specifically, we have provided the mean and standard deviation statistics for every pixel of the GC×GC images across the Macondo samples, as well as the three consecutive injections for the NIST samples. We threshold the pixels of each GC×GC ROI to the parameter Γ, and then measure the highest standard deviation $\sigma_{max}(\Gamma)$, measured at any pixel as the pixel intensity is measured across the sample set (fourteen Macondo samples in Figure S9(a) and three NIST injections in Figure S9(b)).

As expected, the standard deviation decreases while the mean pixel value for the "most variable" pixel increases as we increase the pixel threshold Γ. This is due to the fact that increasing Γ shifts the focus to pixels that represent the target biomarkers which occur in relative abundance and exhibit less chromatographic variability. Please also refer to our discussions on variability and recalcitrance in responses to other reviewers in this regard.

S9.1: Discussion on choice of data portfolio

Successful source differentiation for oil-spill forensics is the primary motivation for this work, and therefore, we provide some key points on why we chose this data portfolio:

(1) We chose samples that would push this method by their likely genetic similarity: Macondo vs. other samples from the Gulf of Mexico (Eugene Island; Southern Louisiana Crude and natural seeped oil), which we expected to be very similar). It would be much easier to highlight disproportionately this method by only investigating oils from around the world where we expected and found much lower PTM scores.

(2) We tested the variability of replicate injections of the same Standard Reference material consecutively within 24 hours to determine the best-case (upper bound) performance of the PTM method for intra-class comparisons.

(3) Every field sample that we collected from the *Deep Water Horizon* (DWH) spill was the same collection on the same day. Grass blade samples were collected shortly afterwards, within a few weeks of the spill samples (Table S1 provides all date details for Macondo samples). They are basically replicate field samples, clearly from the DWH based on visual inspection and on-the-ground knowledge of one of the co-authors, Christopher Reddy. We ran these samples over a multi-day period and observed slightly more variability when compared to consecutive injections.

(4) In our experience in fingerprinting numerous oil spills, the time scale of analysis for this experiment certainly captures the time needed to analyze requisite samples to quantify certainty afforded by this approach.

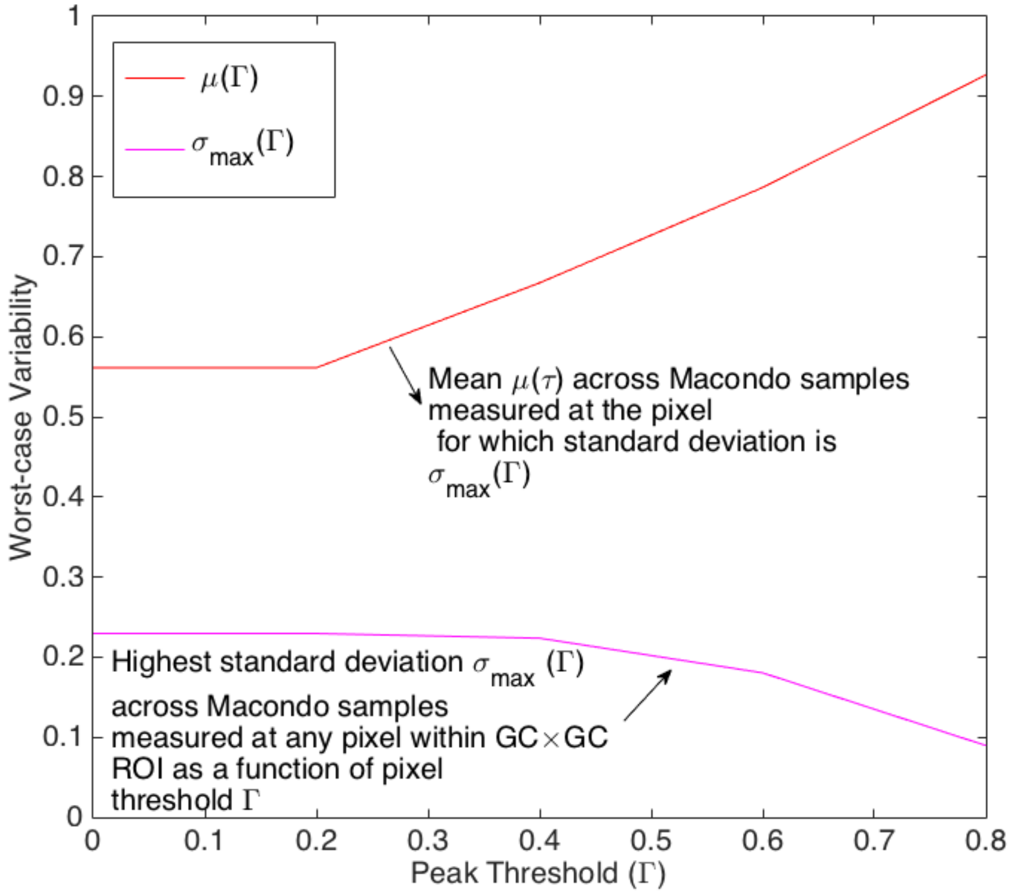

Figure S9(a): Measured variability for GC×GC pixels in the region of interest (ROI), i.e., hopanes and steranes, across (a) fourteen Macondo samples (refer Table S1 for sample details).

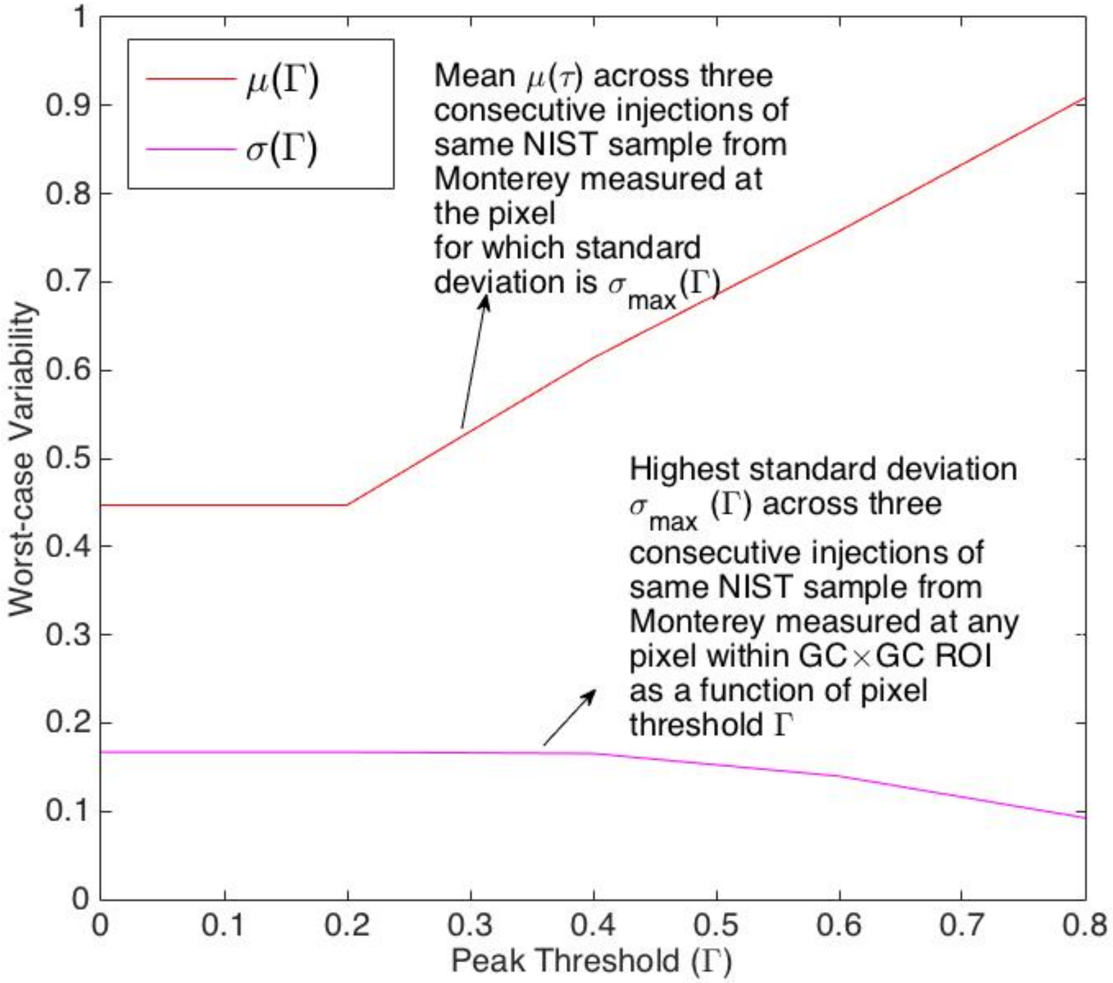

Figure S9(b): Measured variability for GC×GC pixels in the region of interest (ROI), i.e., hopanes and steranes, across three consecutive injections of one NIST sample from the Monterey area (refer Table S1 for sample details).

In synopsis, this method offers an approach for the most challenging oil spill forensics cases, where the challenge comes from inherent regional similarity between oil from sources with geographic proximity, not large separation in collection and run times. Hence, we focused on the “tough” samples to test, e.g. Macondo vs. other Gulf of Mexico sources in the region (See also our point in (1) above). Our target application, i.e., oil spill forensics, typically handles samples that come at the same time due to the nature of the spill event, without significant temporal separation between collection times.
