# Supplementary material for: Interpreting comprehensive two-dimensional gas chromatography using peak topography maps with application to petroleum forensics
Source: Chem Cent J. 2016 Nov 28;10:75. doi: 10.1186/s13065-016-0211-y (PMC5125045; doi:10.1186/s13065-016-0211-y)
Supplement: Supplementary file 11 — Additional file 11: Section S10. Coelution of peaks. [file 13065_2016_211_MOESM11_ESM.pdf]

Section S10: Discussion on coelution of peaks

GC-MS alone does not have the resolving power to provide "baseline" separation of every compound and hence needs to use selective ions. However, GC×GC provides two-dimensional data that produces highly reproducible chromatograms. Families of biomarkers such as hopanes, steranes, and diasteranes elute in fairways on the GC×GC base-plane that are distinct and unique to the precise physical attributes of each biomarker molecule. Hence, co-elution does not occur to the level experienced by GC-MS users. Furthermore, we are confident that our GC×GC-FID peak identifications are accurate as we used numerous biomarker standards (pure biomarker compounds obtained from Chiron and NIST) and utilized the elution positions of each standard compound for the identification of biomarkers in the crude oil samples presented in this manuscript.

Nevertheless, although no mass spectral data was presented, we also utilized peak pattern matching and mass spectral data between GC×GC-TOF and GC×GC-FID instruments to confirm our biomarker identifications. But we prefer GCxGC-FID as the signal is essentially unity for typical petroleum hydrocarbons (see Nelson et al 2016) and hence the signal plane represents an accurate perspective on the relative amounts of material used for PTM. If we had used GCxGC-TOF-MS and total-ion-currents, the responses would be too varied and far from representative.

Furthermore, the National Institute of Standards and Technology and the Gulf of Mexico Research Initiative recently completed an intercomparison on the analysis of Macondo well oil and a weathered oil from the Deepwater Horizon that included 20 laboratories. Our laboratory participated by entering results from GCxGC-FID and they were quite similar to the laboratories that used GC-MS (NIST, 2016)."

The PTM method does not attempt to deconvolve peaks that coelute, but considers peak summits from peaks that may be partially overlapping as separate entries into the PTM representation. As such, the PTM method of forensic interpretation does not compete with prior processing techniques such as deconvolution of coeluted peaks. We performed baseline correction as indicated in the manuscript and supplemental documentation to ensure valid comparison between GC×GC images with different baseline signal strengths. But we have avoided using prior processed data as we wished to examine the “raw” unfiltered performance of the PTM approach unbiased by any pre-processing techniques. PTM interpretation can always be applied to prior processed data, where deconvolution

techniques may be applied.

The PTM method does not interpret based on peak volume, and uses a local maxima finding algorithm to compute the peak height at each peak summit. Therefore, two compound peaks that may co-elute with distinct peak summits will map to distinct PTM nodes.

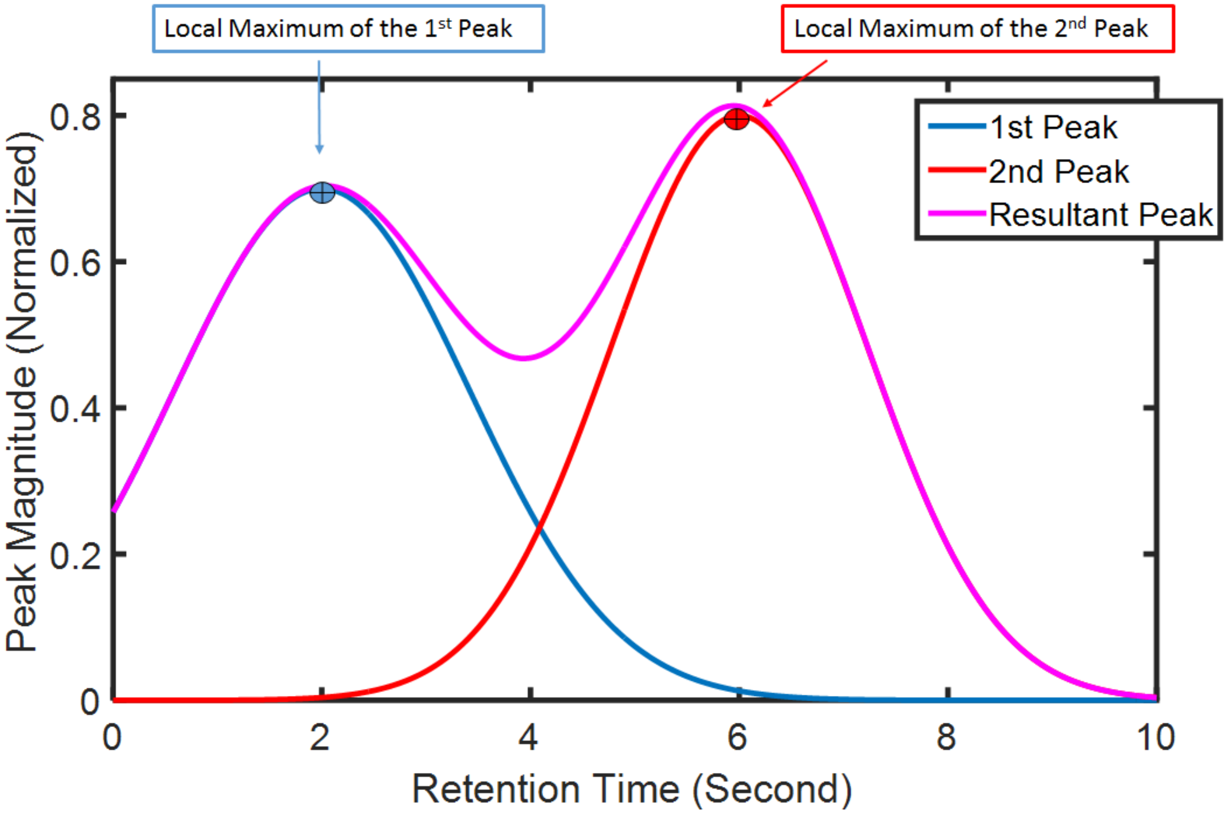

Figure S10. Schematic representation of peak overlap between two coeluting compounds.

Figure S10 illustrates this visually through a schematic diagram. Two compounds shown as blue and red peaks coelute to create the observed peak shown in magenta. Despite overlap between the curves of the first and the second peaks representing the two compounds, the peak summits, shown as red and blue crosses are distinct in this scenario. A local maxima search using gradient calculator can isolate the individual peak summits, and the cross-PTM score matching algorithm (Section S3) uses the peak height, rather than peak volume as the metric for measuring relative abundance.

References:

Nelson, R.K., Aeppli, C., Arey, J.S., Chen, H., de Oliveira, A.H.B., Eiserbeck, C., Frysinger, G.S., Gaines, R.B., Grice, K., Gros, J., Hall, G.J., Koolen, H.H.F., Lemkau, K.L., McKenna, A.M., Reddy, C.M., Rodgers,
R.P., Swarthout, R.F., Valentine, D.L., White, H.W., Handbook of Oil Spill Forensics – Chapter 8 - Applications of Comprehensive Two-dimensional Gas
Chromatography (GC×GC) in Studying the Source, Transport, and Fate of Petroleum Hydrocarbons in the Environment. Zendi Wang and Scott A. Stout,
Editors Copyright Ó 2016, Elsevier Inc.

Murray, J.A., Sander, L.C., Wise, S.A. and C. Reddy. (2016) Gulf of Mexico Research Initiative 2014/2015 Hydrocarbon Intercalibration Experiment: Description and Results for SRM 2779 Gulf of Mexico Crude Oil and Candidate SRM 2777 Weathered Gulf of Mexico Crude Oil. NIST Internal Report 8123. National Institute of Standards and Technology, Gaithersburg, MD. Available at http://dx.doi.org/10.6028/NIST.IR.8123
